# Exploring the theranostic potentials of miRNA and epigenetic networks in autoimmune diseases: A comprehensive review

**DOI:** 10.1002/iid3.1121

**Published:** 2023-12-29

**Authors:** Sagnik Nag, Oishi Mitra, Garima Tripathi, Souvik Samanta, Bikramjit Bhattacharya, Priti Chandane, Sourav Mohanto, Vino Sundararajan, Sumira Malik, Sarvesh Rustagi, Suraj Adhikari, Aroop Mohanty, Darwin A. León‐Figueroa, Alfonso J. Rodriguez‐Morales, Joshuan J. Barboza, Ranjit Sah

**Affiliations:** ^1^ Department of Bio‐Sciences School of Bio‐Sciences & Technology, Vellore Institute of Technology Vellore Tamil Nadu India; ^2^ Integrative Multiomics Lab School of Bio‐Sciences & Technology, Vellore Institute of Technology Vellore Tamil Nadu India; ^3^ Department of Applied Microbiology Vellore Institute of Technology (VIT) Tamil Nadu India; ^4^ Department of Biochemistry School of Life Sciences University of Hyderabad Hyderabad Telangana India; ^5^ Department of Pharmaceutics Yenepoya Pharmacy College & Research Centre Yenepoya (Deemed to be University) Mangaluru Karnataka India; ^6^ Amity Institute of Biotechnology Amity University Jharkhand Ranchi Jharkhand India; ^7^ University Centre for Research and Development University of Biotechnology, Chandigarh University, Gharuan Mohali Punjab; ^8^ School of Applied and Life Sciences Uttaranchal University Dehradun Uttarakhand India; ^9^ Manipal College of Medical Sciences Pokhara Nepal; ^10^ Department of Clinical Microbiology All India Institute of Medical Sciences Gorakhpur Uttar Pradesh India; ^11^ Facultad de Medicina Humana Universidad de San Martín de Porres Chiclayo Peru; ^12^ Clinical Epidemiology and Biostatistics, School of Medicine Universidad Científica del Sur Lima Peru; ^13^ Gilbert and Rose‐Marie Chagoury School of Medicine Lebanese American University Beirut Lebanon; ^14^ Escuela de Medicina Universidad César Vallejo Trujillo Peru; ^15^ Department of Clinical Microbiology Institute of Medicine, Tribhuvan University Teaching Hospital Kathmandu Nepal; ^16^ Department of Clinical Microbiology Dr. D. Y. Patil Medical College, Hospital and Research Centre, Dr. D. Y. Patil Vidyapeeth Pune India; ^17^ Department of Public Health Dentistry Dr. D.Y. Patil Dental College and Hospital, Dr. D.Y. Patil Vidyapeeth Pune Maharashtra India

**Keywords:** autoimmune diseases, diagnostics, epigenetic regulation, miRNA, therapeutics

## Abstract

**Background:**

Autoimmune diseases (AD) are severe pathophysiological ailments that are stimulated by an exaggerated immunogenic response towards self‐antigens, which can cause systemic or site‐specific organ damage. An array of complex genetic and epigenetic facets majorly contributes to the progression of AD, thus providing significant insight into the regulatory mechanism of microRNA (miRNA). miRNAs are short, non‐coding RNAs that have been identified as essential contributors to the post‐transcriptional regulation of host genome expression and as crucial regulators of a myriad of biological processes such as immune homeostasis, T helper cell differentiation, central and peripheral tolerance, and immune cell development.

**Aims:**

This article tends to deliberate and conceptualize the brief pathogenesis and pertinent epigenetic regulatory mechanism as well as miRNA networks majorly affecting five different ADs namely rheumatoid arthritis (RA), type 1 diabetes, multiple sclerosis (MS), systemic lupus erythematosus (SLE) and inflammatory bowel disorder (IBD) thereby providing novel miRNA‐based theranostic interventions.

**Results & Discussion:**

Pertaining to the differential expression of miRNA attributed in target tissues and cellular bodies of innate and adaptive immunity, a paradigm of scientific expeditions suggests an optimistic correlation between immunogenic dysfunction and miRNA alterations.

**Conclusion:**

Therefore, it is not astonishing that dysregulations in miRNA expression patterns are now recognized in a wide spectrum of disorders, establishing themselves as potential biomarkers and therapeutic targets. Owing to its theranostic potencies, miRNA targets have been widely utilized in the development of biosensors and other therapeutic molecules originating from the same.

## 1 INTRODUCTION

Autoimmune diseases (ADs) are chronic and progressive ailments designated by an exaggerated self‐immunogenic response, accompanied by the overproduction of self‐antibodies leading to an overall systemic dysfunction and abnormalities in cellular components. Depending on various biological and physicochemical factors, ADs can bring damage to a particular organ or other biological systems. The interaction of environmental factors and genetic anomalies has a key role in showcasing the pathological effects of Ads.^1^, ^2^ The involvement of B lymphocytes cells in the progression of ADs displays an array of different biological roles. These biological roles mainly include the entrenched secretion of self‐antibodies; the presentation of self‐antigens and arising complementary interactions with T cells; the release of cytokines involved in the inflammatory response; and the development of deranged specialized microstructure named as germinal centers. With the help of these cellular processes, autoimmune conditions that are often categorized as antibody‐mediated or as T cell‐mediated, both are considered to be controlled and affected by B cells.^3^ The maturation of T‐cells in the thymus is responsible for eliminating a large amount of auto‐reactive T cells. However, a bulk of T cells that have matured and can detect autoantigens can be observed in the peripheral circulatory system of healthy people and those suffering from AD. While they appear responsible for the pathophysiology of several ADs in patients, these auto‐reactive cells are maintained in an unresponsive condition in healthy persons. CD4+ CD25+ are considered T cells possessing a natural regulatory mechanism, and furthermore, it is a population of T cells that have been recently discovered and is regarded to be predominantly responsible for the modulation of the activity of these auto‐reactive immune cells.^4^

Recent studies suggest that in some types of autoimmunity, the interaction between the environment and the host is influenced by epigenetic alterations induced by various environmental factors, including altered DNA methylation patterns. Due to environmental factors, it may become difficult for certain cells to maintain epigenetic homeostasis, which can result in loss of tolerance due to abnormal expression of genes. These altered cells can subsequently contribute to the onset of autoimmunity in those with a genetic predisposition.^5^ Epigenetic changes alter the expression of genes and cellular processes, but the genomic sequence remains unaffected. The key epigenetic processes include expression of noncoding RNA, modification of amino termini of histone proteins by posttranslational alterations, and CpG DNA dinucleotides methylation and/or their hydroxymethylationhydroxy methylation. Pathophysiology of ADs has been strongly connected to disease responsible for triggering gene alterations or a combination of genetic vulnerability and epigenetic changes occurring due to various environmental factors. Thus, it is crucial to understand how the concoction of genetic and epigenetic pathways causes some ADs.^6^ A new family of noncoding RNA known as long noncoding RNA (lncRNA) is essential for the control of both autoimmune and immunological processes, whereas, on the other hand, endogenous noncoding RNAs (ncRNAs) known as circular RNAs (circRNAs) showcases itself as the crucial immune system gene modulators and is responsible for the occurrence and progression of ADs.^7^, ^8^ In addition, small, conserved, noncoding RNA molecules called miRNAs target the 3′ untranslated region (UTR) of particular messenger RNAs (mRNAs) and either promote their destruction or suppress translation. Apoptosis, differentiation, cell cycle, and immunological activities are the biological processes that miRNA is known to control. According to recent introspections, miRNAs are essential for the regulatory mechanisms of immunological processes and play a key role in preventing ADs.^9^

The therapy of ADs has changed little over the past few decades due to advancements in medicine, and the mechanisms behind many of these diseases are still unknown. Understanding how ADs initiate, progress, and end is essential. Due to its unique regulatory properties and pathogenic contributions, miRNA can serve as a potential biomarker candidate to efficaciously diagnose AD progression. Several daunting attempts were assembled to construct a compendium of biosensors to detect sole pathogenic miRNA candidates participating in AD pathophysiology. Due to advancing progressions in material sciences and pharmaceutical interventions, several miRNA encapsulating strategies have been formulated to enhance site‐directed specific drug delivery to curb several ADs. Altered physiological microenvironment and physical properties are some of the characteristic hallmarks of AD that demand the application of stimuli‐responsive drug delivery platforms to cater for a stimulus‐specific to the disease. Understanding how miRNAs participate in these processes can offer a new window to advance our knowledge of ADs. This article tends to provide insight into miRNA regulation and responsiveness toward the complexities of immunological cascades associated with progressive ADs, with particular emphasis on rheumatoid arthritis (RA), Type 1 diabetes, multiple sclerosis (MS), systemic lupus erythematosus (SLE), and Inflammatory bowel disorder (IBD), thereby providing optimistic deliberations on novel theragnostic interventions concerning the same. Along the same lines, it also heralds to showcase significant epigenetic modulations for the above‐mentioned ADs.^9^

## 2 BIOGENESIS OF mi‐RNA AND ITS REGULATORY MECHANISM ON ADs

**Figure 1:**
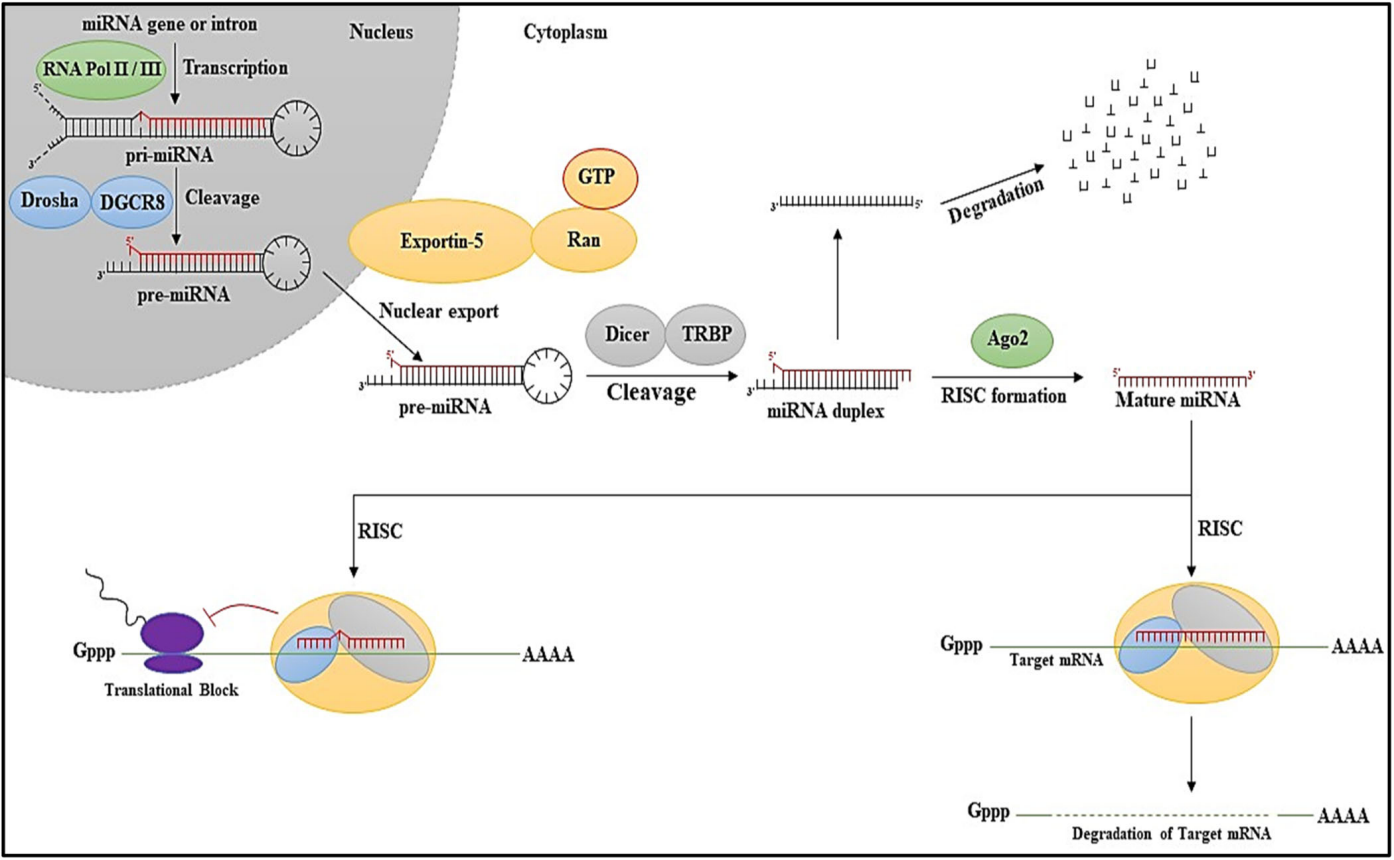
Biogenesis of miRNA. Initial steps include the formation of pre‐miRNA followed by nuclear export and subsequent cleavage to form matured miRNA and further the fate of the matured miRNA is decided by the RISC complex attachment.

Small noncoding RNAs (19–21 nucleotides) called miRNAs^10^ majorly influences the posttranscriptional regulation of gene expression by either limiting messenger RNA (mRNA) translation or encouraging mRNA degradation. MiRNA was first discovered in 1993 and has since been found to be conserved across different species.^11^ miRNAs are the major contributing factors in the pathophysiology of multiple diseases, including cancer, cardiovascular, metabolic, and ADs.^12^ Animal miRNAs are encoded as mono‐cistronic (individual genes), poly‐cistronic (cluster of genes), or introns of host genes (intronic). Primary miRNA (pri‐miRNA) transcripts with hairpins and 5′ and 3′ flanking sequences are produced by RNA polymerase II.^13^ As depicted in Figure 1, the processing is carried out mainly by Drosha and Dicer, two members of the RNase III family of enzymes,^14^ which work in complexes with dsRNA‐binding proteins (dsRBPs), that is, DGCR8 and transactivation‐responsive RNA‐binding protein (TRBP) in mammals, to catalyze the two steps of primary precursor (pre‐miRNA) processing in the canonical pathway.^15^

The structural properties of individual pri‐miRNA sequences influence the effectiveness of pri‐miRNA processing. Co‐transcriptional processing of pri‐miRNAs results in a fast pool of 59‐71‐nt‐long stem‐loop pre‐miRNAs. Exportin‐5, a member of the karyopherin protein family, exports nascent pre‐miRNAs to the cytoplasm in a GTP‐dependent manner.^16^ After penetrating the cytoplasm, pre‐miRNA is processed in the RISC loading complex (RLC) and transformed into a 21‐nt‐long miRNA/miRNA* duplex by ribonuclease Dicer, which is a type III enzyme.^17^ Up to one‐third of human mRNAs may be miRNA targets, and miRNA‐mediated gene regulation is essential for normal physiological processes, including the cell cycle, differentiation, and death. As contemporary research states, miRNAs are essential for controlling immunological processes and evading AD.^9^

There are various checkpoints that guarantee the deletion or silencing of autoreactive T and B lymphocytes, which are produced regularly and randomly throughout lymphomagenesis. However, self‐reactive lymphocytes occasionally get past the checkpoints and continue to live in peripheral lymphoid tissues. When these autoreactive cells are triggered, they launch a vicious assault against self‐tissues that trigger ADs.^18^ miRNAs control autoimmunity by influencing many cell types' formation, differentiation, and function, including innate immune cells (innate immunity), adaptive immune cells (adaptive immunity), and local resident cells.^19^ Toll‐like receptors (TLRs), C‐type lectin‐like receptors (CLRs), nucleotide‐binding oligomerization domain (NOD)‐like receptors, retinoic acid‐inducible gene (RIG)‐I‐like receptors (RLRs) are all expressed by host cells. These receptors can recognize various pathogen‐associated molecular patterns (PAMPs). These processes activate intracellular signaling pathways, resulting in the release of pro‐inflammatory cytokines, chemokines, and interferons (IFNs), as well as the production of co‐stimulatory molecules. Several investigations have demonstrated that miRNAs play critical roles in the biological processes of these adaptive immune cells in autoimmunity; miRNAs also alter/regulate a particular subgroup of T cells called regulatory T cells (Tregs) are essential for regulating the immune response, which finally results in the upkeep of self‐tolerance and homeostasis.^20^ In addition, miRNAs impact the development of CD8+ T cells, Th1 cells, Th2 cells, and Thymus by affecting miRNA‐155, miRNA‐147, and miRNA‐146 levels. The multimodal applications of miRNA delve deep into the conceptualization and understanding of various novel developmental strategies for treating and preventing ADs.^21^

## 3 miRNA IN THE PROGRESSION OF ADs

**Figure 2:**
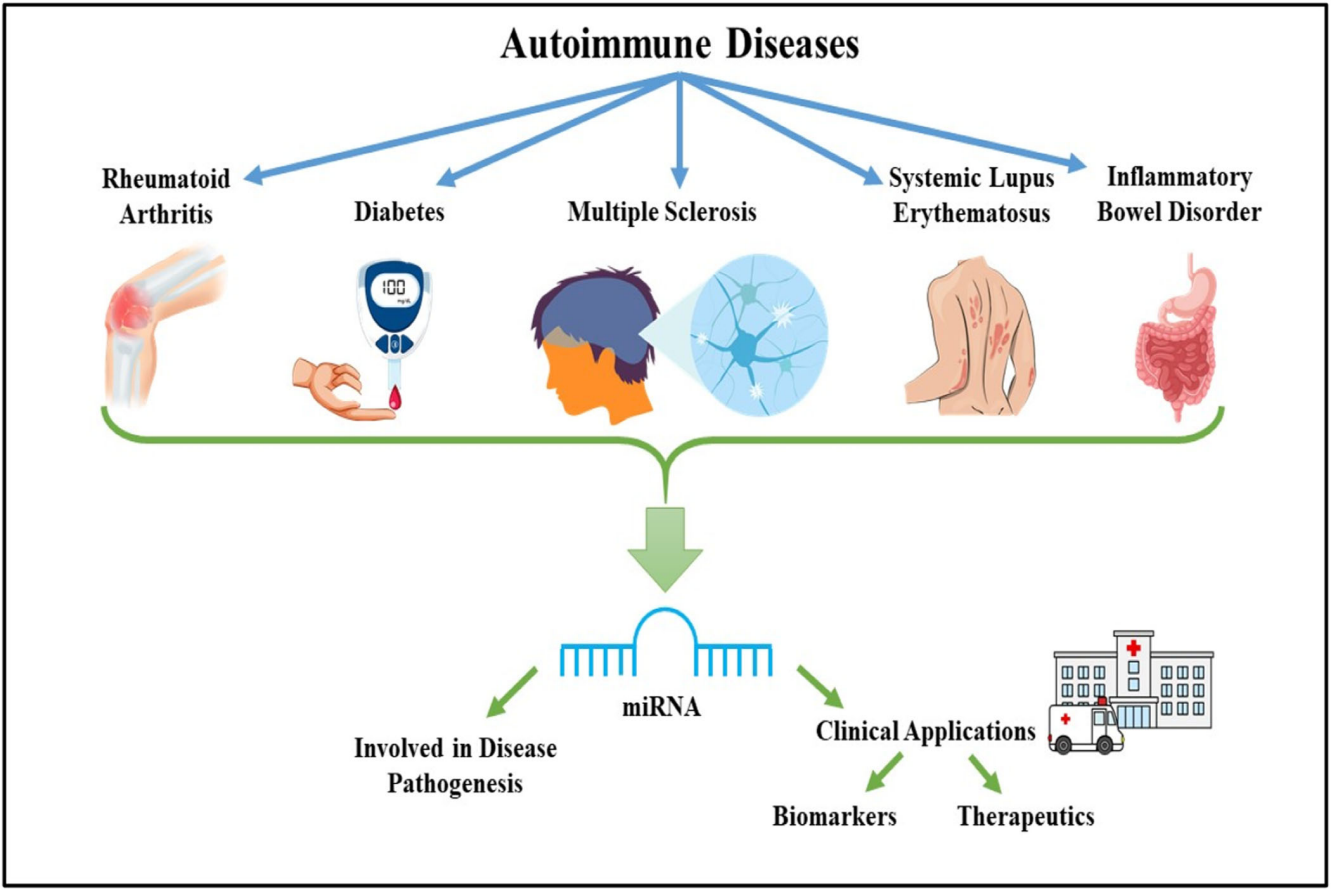
Overview of role of miRNA involved in the disease pathogenesis, biomarker detection and development of miRNA‐based therapeutics for different ADs. ADs, autoimmune diseases.

The miRNAs have always been considered significant contributors to the regular immune system functioning, immunological tolerance pathways and autoimmunity. Recent research from clinical investigations and animal models indicates that miRNAs play a role in the aetiology of various ADs, further depicted in Figure 2. Several human ADs have a significant association between their progression and abnormal miRNA expression. In both central and peripheral lymphoid organs, miRNAs play a role in maintaining immunological tolerance and fighting autoimmune diseases.^22^

### 3.1 Rheumatoid arthritis (RA)

**Figure 3:**
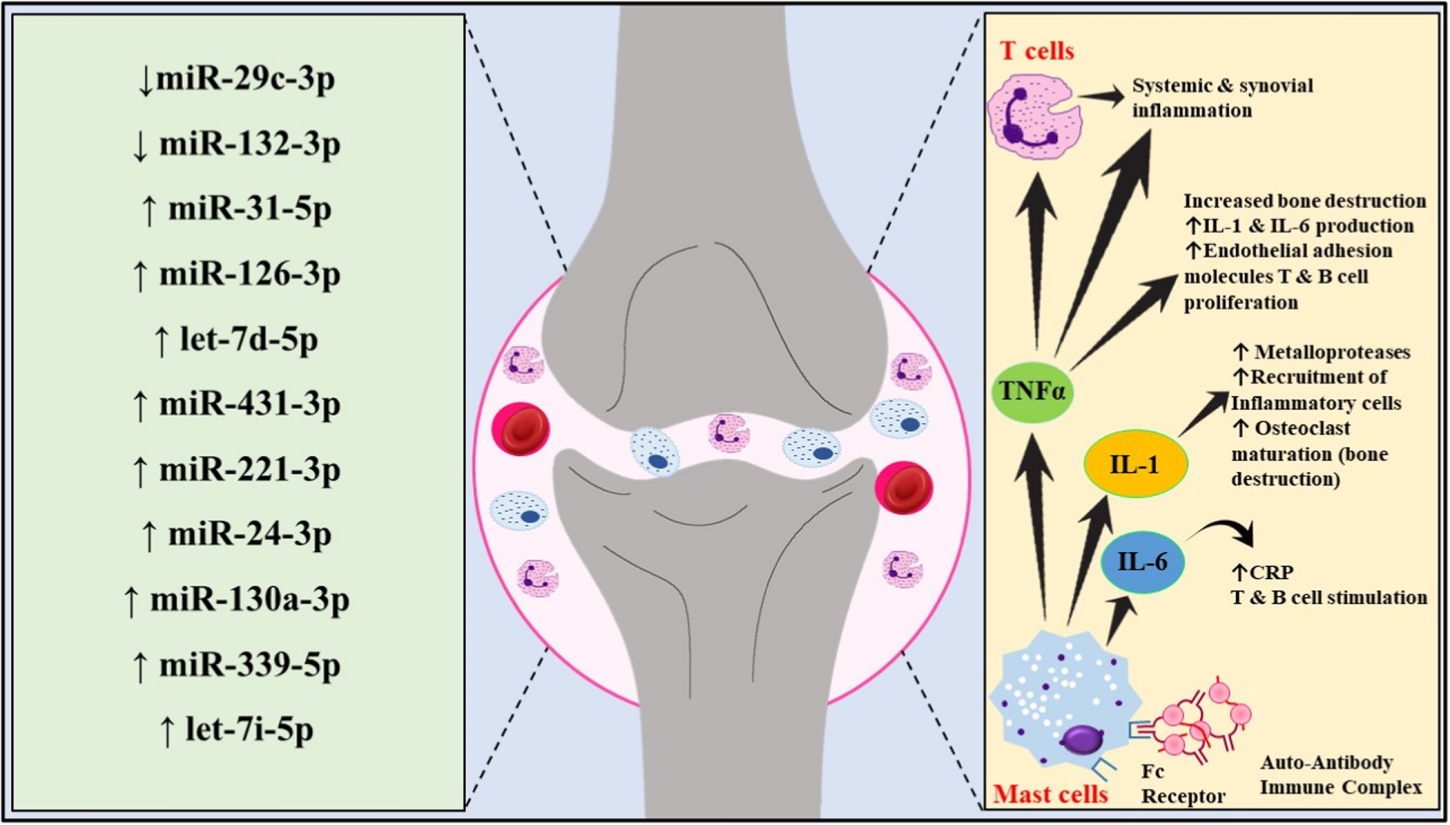
Detailed immune regulation and miRNA networks involved in RA proliferation. Illustrative representation of multifaceted roles of mast cells secreting several pro‐inflammatory interleukins like IL‐6, IL‐1, and tumor necrosis factors leading to osteoclast maturation and subsequent bone degradation. RA, rheumatoid arthritis.

RA is a debilitating, complex autoimmune disease. It causes persistent musculoskeletal pain, prolonged morning stiffness, and inflammation of the tissues surrounding synovial joints, cartilage, and bones. If left untreated, it can lead to bone erosion and joint damage.^23^, ^24^ This symmetrical AD also affects the internal extra‐articular organs like skin, eyes, lungs, heart, kidney, and blood vessels.^24^, ^25^ People afflicted with RA range from 0.24% to 1% globally.^26^ It is predominant in women compared to men and has increased prevalence in the age group of 50–65 years.^27^ The emergence of noncommunicable diseases has become a leading cause of mortality worldwide, and RA is one of them. These diseases severely affect both developed and low‐middle‐income countries (LMICs).^28^ RA being a complex AD, the pathophysiology is regulated by several inflammatory markers and immunogenic proteins. B lymphocytes, T lymphocytes and macrophages predominantly contribute to the progression of the disease.^29^ These autoreactive B cells induce the secretion of cytokines like tumor necrosis factor‐α (TNF‐α), interleukin‐6 (IL‐6), IL‐12, IL‐23, and IL‐1α in the diseased tissue. Another cytokine receptor activator of nuclear factor κB ligand (RANKL) has been found to show enhanced production from memory B lymphocytes in peripheral blood, synovial fluid, and affected tissue of patients. This cytokine is responsible for bone resorption as it binds to its receptor and stimulates differentiation and activation of osteoclasts.^30^ In addition, B lymphocytes are also responsible for the secretion of rheumatoid factors (RF) and anticitrullinated protein antibodies (ACPA).^31^ Figure 3 further summarized the immune regulation and various miRNA networks involved in RA proliferation.

The activation of T lymphocytes is stimulated by both B cells and macrophages. Synovial T cells have been reported to secrete cytokines like IL‐15, IL‐7, IL‐13, IL‐4, IL‐2, essential fibroblast growth factor, and epidermal growth factor, and these cytokines have a significant contribution in the aetiology and pathogenesis of the ailment.^30^ In the early stages of the disease, macrophages stimulate T cells and enhance the production of IL‐1α, IL‐1β, and Matrix metalloproteinases (MMPs).^31^ The inflammatory mediators secreted by the autoreactive immune cells interact with neurons and neuroglia, resulting in neurogenic inflammation.^31^ RA patients are often broadly classified into ACPA‐positive and ACPA‐negative. These two patient types have very different pathophysiologies, ACPA‐positive being a more clinically aggressive phenotype. Citrullination is categorized as a posttranslational modification that synthesizes a polar (neutral) citrulline from a positively charged arginine and is also aided by Ca2+‐requiring enzyme peptidyl arginine‐deiminase (PAD). The genetic and epigenetic factors interact and trigger the production of ACPA in diseased individuals. Environmental factors like smoking and inhaling nanosized silica or silica dust may trigger mucosal‐TLRs, which activate Ca2+‐dependent PADs and antigen‐presenting cells (APCs) like dendritic cells and B lymphocytes.^32^

The miRNA‐117‐5p expression is seen to be reduced in chronic RA. This miRNA explicitly targets the JAK‐STAT pathway and downregulates the IL6 expression. Similarly, several miRNAs act as a biomarker and can be detected from the synovial fluid or tissue in RA patients. miRNA‐34a is regulated by the NF‐kB family of cell signaling molecules, which are further activated by inflammatory cytokine molecules.^33^ miRNA‐29a acts as an anti‐inflammatory marker in RA fibroblast‐like synoviocytes (RA‐FLSs). The drop in miR‐199a‐3p expression contributes to local hyperplasia. miRNA‐375 in RA‐FLSs exhibits a positive effect on synovial pathogenesis due to canonical Wnt signaling inactivation, thereby influencing the progression of the disease and severity of the condition.^34^ Exosome‐secreted miRNAs are important biomarkers and are vital targets for developing therapies to treat RA. According to a study by Cunningham and colleagues, eight serum miRNAs viz., miRNA‐126‐3p, let‐7d‐5p, miRNA‐431‐3p, miRNA‐221‐3p, miRNA‐24‐3p, miRNA‐130a‐3p, miRNA‐339‐5p, let‐7i‐5p are found to have enhanced expression in RA afflicted individuals compared to that of healthy individuals.^35^ Exosome‐derived miRNA‐486‐5p from synovial tissue is associated with tumor initiation or suppression in several cancers and pulmonary fibrosis. miRNA‐486‐5p secreted from the exosomes of RA Fibroblast‐like synoviocytes (RA‐FLS‐Exo) has been reported to promote osteoblast differentiation in RA patients by decreasing the expression level of Tob1 gene and triggering the bone morphogenetic protein (BMP)/Smad pathway.^36^ Fibroblast‐like synoviocytes are known to be responsible for cartilage destruction and maintenance and progression of inflammation in RA patients. Mesenchymal stem cell (MSC)‐derived exosomes are evident to exhibit immunosuppressive effects like suppressing B and T lymphocytes. miRNA‐320a is found to have deficient expression levels in RA and is known to have an antiproliferative effect and increase the apoptotic death of FLSs by downregulating MAPK/ERK1/2 signaling pathway.^37^ There are CD4+CD25+Foxp3+ T cells, also called regulatory T lymphocytes or Treg cells. These cells are known to inhibit the autoimmune response found in RA. Patients nonrespondent to disease‐modifying antirheumatic drugs (DMARDs, or Methotrexate) are often treated with biological DMARDs or immunotherapy. Increasing the number of Treg cells has demonstrated a reduction in the pro‐inflammatory responses. Stimulation of Treg cell‐specific targeted gene proliferation induced more and more production of Treg cells, which slowed the disease progression and proved to be an essential treatment for ADs.^38^

### 3.2 Type I diabetes

Diabetes is a complicated and persistent clinical condition requiring continuous biomedical support and preventive measures that are not concerned with glucose management.^39^ Three conditions may classify diabetes as Type 1 diabetes (T1D), Type 2 diabetes (T2D), and gestational diabetes.^40^ T1D, out of the above, is concerned with an autoimmune abnormality wherein the β‐cells producing Insulin in the pancreas are destroyed.^41^ The occurrence of diabetes in adults varying in the age group of 21–80 over the world was predicted to shoot up to 10.6% (536.7 million) in 2021 and 12.3% (783.3 million) in 2045.^42^

Small endogenous miRNAs are predisposed to control cellular cycles, proliferation, differentiation, and apoptosis by inhibiting the biogenic molecules that control these processes, such as RNA transcripts, or by degrading mRNA.^43^ According to various research groups, miRNA‐375 was upregulated, impacting the beta cells that secrete insulin and other miRNA molecules such as miRNA‐29a, miRNA‐29b, miRNA‐200, and miRNA‐7.^44^ A cross‐sectional nested, case‐control‐based research study was carried out by Barutta and colleagues, to look at the differential expression of miRNAs in the blood samples of patients suffering from T1D. A considerable upregulation of the miRNAs, that is, miRNA‐140‐3p, miRNA‐574‐3p, miRNA‐139‐5p, miRNA‐106a, miRNA‐17, miRNA‐486‐3p, miRNA16, miRNA‐222, and miRNA‐885‐5p was reported.^45^ Children with new‐onset T1D were found to have blood samples significantly overexpressed miRNA‐197. It was also claimed that miRNA‐197 can accurately predict residual beta‐cell functioning.^46^ miRNA‐155, miRNA‐92a, and miRNA‐126 were noticeably downregulated in the blood samples of T1D patients.^45^ Additionally, T1D is frequently characterized by endothelial homeostasis maintained by miRNA‐126. Additionally, miRNA‐126 regulates endothelial inflammation in individuals with micro/macrovascular problems related to T1D, establishing a connection between these issues and reduced levels of miRNA‐126.^43^

The NF‐κB response triggered by lipopolysaccharide in monocytes and dendritic cells serves as a phenotypic characteristic and a potential indicator of pathogenic processes in human T1D.^47^ In animal murine models of T1D, it has been observed that TNF receptor‐associated factor 6 (TRAF6) plays a mediating role in endothelial damage induced by high glucose levels, and this process is governed by signaling pathways involving NF‐κB and AP‐1.^48^, ^49^ In human monocytic lymphocytes, miRNA‐146a, a transcriptional product of the LOC285628 gene situated on the 5th chromosome, is responsive to lipopolysaccharide (LPS) stimulation. Its activation hinges upon the nuclear factor kappa B (NF‐κB). The putative role of miRNA‐146a is contingent upon this signaling cascade, wherein its heightened expression via NF‐κB signaling results in its capacity to orchestrate the downregulation of target genes, such as TRAF6 and IL‐1 receptor‐associated kinase 1 (IRAK1). This, in turn, leads to the moderation or cessation of an exaggerated inflammatory response through a regulatory negative feedback loop.^50^, ^51^ A study conducted by Gomez‐Muñoz and colleagues, envisaged the immunomodulatory potencies of miRNA‐30d‐5p in the remission phase of T1D regulating PD‐1 expression and manifesting T‐Cell Expansion. Upon small RNA sequencing and in‐vivo evaluation, it was observed that lower levels of miRNA‐30d‐5p elevated regulatory T‐cell populations around pancreatic lymph nodes coupled with an elevated expression of CD200, in addition to a decrease of PD‐1+ T lymphocytes in the spleen.^52^

Among microRNAs that were analyzed in more than one study, about 21 (miR‐15b, miR‐20b‐5p, miR‐22‐3p, miR‐21‐5p, miR‐25‐3p, miR‐24‐3p, miR‐26b‐5p, miR‐27b‐3p, miR‐100‐5p, miR‐148a‐3p, miR‐146a‐5p, miR‐181a‐5p, miR‐150‐5p, miR‐200c‐3p, miR‐210‐5p, miR‐335‐5p, miR‐375, miR‐342‐3p, miR‐1275, let‐7g‐5p, and let‐7f‐5p) were found in plasma/serum or T/PBMCs/cells and, have the potential to be circulating biomarkers of T1D.^53^ Upregulated miRNAs can control inflammation by directing genes such as cytokine and immune cell activation signals. MiR‐146a, for example, is known to be increased in T1D and targets inflammation‐related genes such as tumor necrosis factor receptor‐associated factor 6 (TRAF6) and interleukin‐1 receptor‐associated kinase 1 (IRAK1). This can aggravate the inflammatory response and lead to the death of beta cells.^54^ Upregulated MiRNAs can also target genes essential in beta cell function and survival. For example, in T1D, miR‐375 is increased and can inhibit genes involved in insulin production, resulting in poor glucose management.^55^ An alteration to the daily routine, following a holistic diet, biomedical or surgical therapeutic procedures, or considering a combination of these methods can lead to diabetes remission. Lifestyle changes that affect daily activities linked to nutrition and exercise have health impacts beyond those specifically relevant to diabetes.^56^

### 3.3 Multiple sclerosis (MS)

MS is categorized as an auto‐immunogenic inflammatory disease inducing persistent demyelination in the central nervous system (CNS). According to a study conducted between September 2019 and March 2020, it was statistically estimated that 2.8 million individuals across the globe suffer from MS, approximately 35.9 per 100,000 population.^57^ In younger individuals, MS is the predominant contributor to atraumatic neurological impairment.^58^ The clinical features of MS involve dispersed foci across the white matter that reappear throughout its progression.^59^ The etiological factors that lead to the onset of MS include T‐cell‐mediated immune responses that trigger cytokine release, alter the barrier permeability between the brain and cerebrospinal fluid,^60^ axonal inflammation, gradual demyelination and a rise in the pro‐inflammatory miRNAs and its relevant hallmarks. Macrophages, followed by CD8+ T‐cells are significantly observed in the influx, along with a lesser number of CD4+ T cells, B cells, and plasma cells. Also, the lymphocytes that majorly contribute to the progression of MS are Th1 and Th17.^61^

Diverse genetic, epigenetic, microbiological, and ecological variables aid the pathogenesis of MS. miRNAs are quintessential epigenetic phenomena that regulate abnormal cellular events during the prognosis of the disease. The key purpose of miRNA is to monitor and control the translation of genes, either by suppressing translation or cleaving the target mRNA. In MS, miRNA expression profiles are altered in CNS lesions and the immune system, significantly affecting gene expression in the array of cell types and ultimately promoting the disease.^61^ In the study by Baulina and colleagues, a range of miRNAs having altered expression during the disease prognosis from varied sources were outlined, including miRNA‐155, miRNA‐146a, miRNA‐181c, miRNA‐326, miRNA‐346, miRNA‐17, miRNA‐320a, miRNA‐34a, miRNA‐340, miRNA‐132, and their target sequences.^62^ Based on multiple data identified, miRNA‐155 is regarded as the most potent promoter of inflammation and crucial for the pathophysiology of the disease under myeloid cell polarization to a morphological and active pro‐inflammatory type. Upregulation of miRNA‐155 has been detected in the cellular blood samples of individuals affected by MS, which may be a sign of a severe disease progression. miRNA‐155 modulates the MS risk genes PIK3R1 and PIK3CA which encodes for proteins belonging to the phosphoinositide 3‐kinase (PI3K) family. Dysfunction of the PI3K family leads to oncogenesis, neurological and immune disorders, and demyelination in MS.^63^

The dysregulation of miRNA‐3606‐3p in patients of systemic sclerosis is a prominent biomarker and can reduce TGFBR2 expression.^64^ This can lead to the severity of the diseased condition. However, an upsurge in miRNAs like miRNA‐126 and miRNA‐139‐5p in systemic sclerosis has found a correlation to inflammatory cytokines and signaling molecules like IFN‐B.^65^ Concerning a study on miRNAs to ascertain their correlation with gadolinium‐enhancing (Gd+) lesions for evaluating their utility as MS activity biomarkers, it was observed that peripheral blood mononuclear cells (PBMCs) of MS patients had overexpression of miRNA‐146a/b in comparison to the control. They hypothesized that this elevation was particular to the critical stage of MS and contributed to the proliferation of Th cells, which are effective in the aggravating mechanisms that occur in the CNS of MS patients. First, activated T cells traverse through the blood‐brain barrier from the periphery into the CNS. Cytotoxic CD8+ T cells can directly cause axonal damage, while CD4+ T cells aid in retaining the CNS inflamed. Furthermore, regulatory T cells (T reg) that sustain immune tolerance by inhibiting effector T cells carry out this control through the secretion of miRNA‐based exosomes, a route for gene silencing.^66^ miRNA‐20a‐5p and miRNA‐20b‐5p were shown to be critical regulators of 1000 targeted genes according to database analysis of miRNA‐mRNA interactions 46, indicating that they may be key players in MS pathogenesis.^67^ By analyzing the association of the expression of miRNAs and its potential target genes, the involvement of miRNAs in the disease prognosis has been established. Nevertheless, the exact functioning of miRNA in the pathogenesis of MS ought to be reasonably identified. New medications and therapeutic modalities that affect miRNAs will undoubtedly emerge with the development and use of new advances in research, providing the groundwork for the eventual eradication of different immunogenic disorders.^67^

### 3.4 SLE

The SLE is identified as a persistent inflammation associated with auto‐immunogenic ailment, caused by the progressive decline of resistance to the self–antigens, stimulation of dysfunctional immune T cells and B cells, synthesis of autoantibodies (auto‐Abs), and altered cytokine activity. Due to the advent of complex molecular associations between epigenetic triggers, unbalanced hormone levels, genetic susceptibility, epigenetic control, immune status, and other unknown factors, SLE is strongly related to the downregulation of innate and adaptive immunological responses.^68^ miRNAs are significant regulators of ADs, where miRNA‐146a and miRNA‐155 are demonstrated to be crucial in the pathogenesis of SLE. miRNA‐146a was found to be adversely correlated with the expression of the type I IFN regulatory signaling system. The toll‐like receptor (TLR)‐myeloid differentiation factor 88 (MyD88) pathway, which includes IRAK1 and TRAF6, was a critical regulator of signaling pathways.^69^ The miRNA‐155 is verified to upregulate the alteration of Treg phenotype in MRL/lpr mice by modifying the CD62L expression.^70^ miRNA‐17 is associated with the production, division and activation of immune B cells, T helper cells namely Th1, Th2, Th17.^71^ In a study conducted by Kaga and colleagues, miRNA‐17 production was found to be more in the healthy control as compared to that of the SLE patients, and it was also observed that miRNA‐17 has an antagonistic relation with the interferon alpha mRNA that may play a role in the etiology of the disease.^72^

miRNA‐142‐3p was considerably downregulated in SLE patients. miRNA‐142‐3p, primarily produced in hematopoietic stem cells, has been identified as crucial for the immune response, particularly in macrophages and Regulatory T cells.^73^ miRNA‐20a downregulation was linked to lupus nephritis and vascular thrombosis.^74^ miRNA‐125a comprises the inflammatory chemokine pathway. Recent studies have demonstrated that miRNA‐125a targets KLF13 in SLE, which increases the production of the inflammatory chemokine RANTES.^70^ miRNA‐15a has explicitly a deleterious impact on the B‐10 subpopulation, and miRNA‐15a reduction may help treat SLE.^75^ In a scientific study designed by Yan and colleagues, miRNA‐124‐3p and miRNA‐377‐3p were highly expressed in PBMCs and serum collected from patients affected by SLE in comparison to the healthy controls.^76^ miRNA‐21 is upregulated due to the hyperactivity of immune T cells, whereas miRNA‐7 is related to the overproduction of B cells and autoantibodies. miRNA‐34a is responsible for the collapsing of the immunological tolerance.^77^

### 3.5 Inflammatory bowel disorder (IBD)

The term “IBD” corresponds to a group of relapsing, chronically inflammatory gastrointestinal illnesses, notably Crohn's Disease and Ulcerative Colitis.^78^ Over the past decade, as lifestyles and dietary practices became more westernized, IBDs expanded worldwide.^79^ According to experimental data, excessive consumption of certain macronutrients in the modern food habit triggers an inflammatory response in the colon by preying on innate immune sensors and disrupting the metabolism of gut microbes. Even though incidence is stabilizing in Western nations, the burden is still significant since prevalence exceeds 0.3%. These findings underline the need for research into IBD prevention and advances in healthcare systems to manage this complicated and expensive condition. Immunometabolism regulates vulnerability to intestinal inflammation and the risk of IBD genetically. This partially influences metabolism and stress‐related signaling of innate immunity.^80^ Some micro‐RNAs, including miRNA‐101, miRNA‐515‐5p, miRNA‐623, miRNA‐325, miRNA‐876‐5p, miRNA‐1224‐5p, miRNA‐1226‐5p, and miRNA‐1253, have been found to invade bacterial membrane and subsequent regulate gene transcription. This promotes bacterial proliferation and mobility, affecting the population and diversity of the gut microorganisms.^81^

The most often researched miRNAs in relation to IBD appear to be miRNA‐21, miRNA‐155, and miRNA‐31.^82^ miRNA‐21 is prevalent in IBD due to its links to the disease and functional relevance in mice models. Additionally, miRNA223 regulates innate immunity in intestinal inflammation.^83^ miRNA 214‐3p and miRNA 206 activate the NF‐kB pathway and promote intestinal inflammation. Some members of the regenerative gene (REG) family, including (REG Iα, REG Iβ, and REG IV) are expressed in Crohn's disease and ulcerative colitis and have a role in proliferative mucosal components in IBD. Through downregulating miRNA‐24, LPS caused REG IV expression in human intestinal epithelial cells. Intestinal epithelial cells' RAGE/TLR4 receptors controlled the LPS signal.^84^ By reducing the overexpression of inflammatory cytokine receptors like IL7R and IL17RA as well as signal proteins like GP130, miRNA‐31 reduced the inflammatory response in a mouse model of Dextran Sodium Sulfate‐induced colitis, which Tian and colleagues, 2019 found to be hyper‐expressed in tissues from patients suffering from IBD.^85^ Another study using the DSS mouse model revealed that miRNA‐155 binds to SHIP‐1 mRNA directly, resulting in a considerable drop in SHIP‐1 levels, which control cell membrane trafficking.^86^

Crohn's disease, also called Crohn's syndrome, is a persistent inflammatory bowel illness that affects the digestive tract. Common symptoms include diarrhea, stomach discomfort, exhaustion, weight loss, a lack of appetite, bloody stool, fever, joint stiffness, skin issues, mouth sores, perianal sensations, and vision difficulties. Diarrhea can be severe and contain blood or excessive mucus. Abdominal aches and cramps are also possible. Chronic inflammation can cause fatigue and weakness. Joint discomfort can mimic arthritis, and skin disorders such as rashes, blisters, or ulcers might develop. Perianal symptoms might result in painful and inconvenient internal fissures or fistulas. This disease is a complicated inflammatory bowel condition with miRNA patterns that can aid understanding and lead to novel treatment approaches. MiRNA‐21, an upregulated miRNA that inhibits anti‐inflammatory factors, is linked to inflammation.^87^ Another increased miRNA, MiR‐155, is involved in immune system regulation and inflammation.^88^ MiR‐192, a downregulated miRNA, was shown to be downregulated in Crohn's disease patients, indicating a possible role in intestinal epithelial barrier function. Another downregulated miRNA, MiR‐31, has been discovered to be decreased in inflammatory colonic mucosa, perhaps leading to aberrant tissue repair. Understanding these miRNA profiles can aid in developing novel Crohn's disease therapeutics.^89^

MiR‐21 suppresses anti‐inflammatory molecules, causes inflammation via many mechanisms. It specifically targets and suppresses the expression of PTEN, a negative regulator of the NF‐B signaling pathway, resulting in enhanced NF‐B pathway activation and the generation of pro‐inflammatory cytokines and chemokines.^90^ It also suppresses the expression of SOCS3, a negative regulator of the STAT3 signaling pathway that promotes the synthesis of pro‐inflammatory cytokines and chemokines, in addition to cell proliferation and survival. Furthermore, miR‐21 suppresses autophagy, a mechanism that eliminates injured cells and cellular detritus, resulting in the buildup of pro‐inflammatory chemicals and the development of inflammation. It also alters the gut microbiome, which is a bacterial colony in the intestinal tract that regulates inflammation. This can lead to the proliferation and survival of inflammatory cells, leading to an excessive accumulation of inflammatory cells in the gut which contributes to the inflammation.^91^ MiR‐31 expression is reduced in the colon mucosa, resulting in aberrant tissue repair. This suppression disturbs a number of critical pathways and activities, including inflammation, proliferation of epithelial cells and differentiation, and autophagy. MiR‐31 inhibits inflammatory signaling by downregulating the production of pro‐inflammatory cytokines and signaling molecules such as IL‐6, IL‐17, and STAT3. This aids in the suppression of inflammation and the healing of wounds. MiR‐31 also regulates epithelial cell proliferation and differentiation by downregulating the activity of genes involved in these processes.^85^ Axin1, Lats1/2, and ATG16L1 are among these genes. Autophagy is a biological process that eliminates damaged proteins and organelles from the cell to preserve cellular homeostasis and avoid cell death. Downregulation of MiR‐31 can cause deregulation of numerous pathways and activities, contributing to abnormal tissue repair and the occurrence of cancer. STAT3 is a gene transcription factor involved in inflammatory signaling. MiR‐31 binds to the 3′‐UTR of STAT3 and inhibits its expression, decreasing inflammation and facilitating wound healing. MiR‐31 inhibits IL‐6, a pro‐inflammatory cytokine, by binding to its 3′‐UTR. MiR‐31 regulates epithelial cell proliferation and differentiation by downregulating the levels of Axin1, Lats1/2, and ATG16L1. This ensures that the epithelial cells multiply and differentiate at a suitable pace to heal injured tissue.^85^, ^91^

Ulcerative colitis is another type of intestinal inflammation that primarily affects the colon and rectum over time. Diarrhea, stomach discomfort, rectal bleeding, urgency and incomplete bowel movements, loss of appetite, weight loss, exhaustion, vision difficulties, joint pain, skin disorders, mouth sores, and fever are all possible symptoms.^92^ Researchers discovered many elevated or downregulated miRNAs in ulcerative colitis (UC) patients, which can contribute to disease pathogenesis by altering genes that regulate inflammation, immunological responses, and repair of tissues. MiRNAs that are upregulated include miR‐223, miR‐21, miR‐192, miR‐155, miR‐146a, miR‐126, miR‐375, and the miR‐30 family. MiR‐21 induces inflammation by regulating the NF‐B signaling pathway and targeting anti‐inflammatory molecules.^87^ MiR‐155 modulates the immune system's reaction and has been associated with increased inflammatory cytokine production.^93^ MiR‐223 is linked to UC and has a role in aberrant immunological responses. MiR‐192 can potentially affect epithelial barrier functioning and inflammation in the colon.^94^ Downregulated miRNAs include members of the miR‐146a, miR‐375, miR‐126, and miR‐30 families. MiR‐146a promotes mucosal integrity and immunological responses, whereas miR‐375 regulates mucosal integrity and immune responses. MiR‐126 mediates dysfunction of endothelial cells and vascular abnormalities associated with UC.^95^

MiRNA155 regulates multiple molecular pathways involved in UC. These pathways include NF‐B signaling, which promotes colon inflammation, and STAT3 signaling, which promotes the development of UC. MiRNA155 can potentially promote inflammation and tissue damage by suppressing apoptosis in colonic epithelial cells. Furthermore, miRNA155 has the ability to block autophagy, a process in cells that eliminates damaged organelles and proteins, which contributes to inflammation and tissue damage. MiRNA155 can also directly target genes implicated in UC, such as SOCS1 and SHIP1, that are JAK/STAT signaling negative regulators. It increases STAT3 signaling and inflammation by targeting and inhibiting SOCS1 expression.^96^ MiRNA‐155 stimulates the synthesis of cytokines that promote inflammation such as tumor necrosis factor‐alpha, interleukin‐6, and interleukin‐8 by targeting SOCS1. Furthermore, miRNA155 has the ability to target and decrease SHIP1 expression, resulting in enhanced PI3K/AKT signaling and proliferation of cells. miRNA155 has also been shown to inhibit FOXO3a expression, promoting apoptosis and inflammation.^97^ MiRNA192 is essential for the correct functioning of the colon's epithelial barrier and inflammation, further, affects the operation of tight junctions, which form seals between neighboring epithelial cells and prevent undesirable molecules from entering the circulation. MiRNA192 inhibits the production of tight junction proteins such claudin‐1 and occludin, resulting in enhanced paracellular permeability.^98^ It also alters the inflammatory reaction via downregulating pro‐inflammatory cytokines like IL‐6 and TNF‐ and upregulating anti‐inflammatory cytokines like IL‐10, implying that it may protect against colon inflammation. MiRNA192 specifically targets the RhoA/ROCK signaling pathway, which plays a role in a variety of physiological functions such as cell migration, contraction, and adhesion. It inhibits RhoA and ROCK expression, resulting in enhanced epithelial barrier permeability. Furthermore, miRNA192 inhibits the production of NF‐kB, an important regulator of the response to inflammation. This downregulation may result in less inflammation in the colon. RhoA and ROCK are crucial in tight junction integrity, and miRNA192 may suppress their expression by attaching to their mRNAs and inducing degradation. This increases the permeability of the epithelial barrier. The transcription factor NF‐kB is important in the inflammatory response because it activates the production of pro‐inflammatory genes. MiRNA192 can suppress NF‐kB production by binding to its mRNA and increasing its breakdown, hence lowering colon inflammation^99^ (Table 1).

**Table 1:** Different ADs and their respective miRNAs associated with disease pathogenesis.

Autoimmune disease	miRNA	Regulation	Mechanism	References
Rheumatoid arthritis	miRNA‐126‐3p, let‐7d‐5p, miRNA‐431‐3p, miRNA‐221‐3p, miRNA‐24‐3p, miRNA‐130a‐3p, miRNA‐339‐5p, let‐7i‐5p, miRNA‐486‐5p	Upregulated	Promotes apoptosis and pro‐inflammatory markers. Antiproliferative effect and increases the apoptotic death of FLSs. Maintains homeostasis and associated with cartilage development pathways.	[34, 100, 101, 102, 103]
	miRNA‐320a, miRNA‐132, miRNA‐363, miRNA‐498a, miRNA‐124a, miRNA‐140, let‐7a, miR‐204‐5p	Downregulated		
Type I diabetes	miRNA‐375, miRNA‐29a, miRNA‐29b, miRNA‐200, miRNA‐7, miRNA‐140‐3p, miRNA‐574‐3p, miRNA‐139‐5p, miRNA‐106a, miRNA‐17, miRNA‐486‐3p, miRNA16, miRNA‐222, miRNA‐885‐5p, miRNA‐197	Upregulated	Malabsorption of glucose and insulin resistance. Maintains glucose homeostasis. Beta cell differentiation and proliferation, regulates apoptosis. Controls glucose and lipid metabolism.	[104, 105, 106, 107]
	miRNA‐155, miRNA‐92a, miRNA‐126, miRNA‐34a, miRNA‐214‐3p miRNA‐27‐3p	Downregulated		
Multiple sclerosis	miRNA‐155, miRNA‐146a/b, miRNA‐214, miRNA‐23a, miRNA‐219, miRNA‐338, miRNA‐128, miRNA‐27b, miRNA‐340, miRNA‐29b, miRNA‐326, miRNA‐301a, miRNA‐17‐5p	Upregulated	miRNA expression altered in CNS lesions and in the immune system, which effects gene expression and promotes the disease. Contribute toward Th‐17 differentiation and polarization.	[108, 109, 110, 111, 112]
	miRNA‐219, miRNA‐338, miRNA‐23b, miRNA‐25, miRNA‐15a, miRNA‐16‐1 miRNA‐17‐92 cluster	Downregulated		
Systemic lupus erythematosus	miRNA‐155, miRNA‐15a, miRNA‐124‐3p, miRNA‐377‐3p, miRNA‐21, miRNA‐7, miRNA‐34a, miRNA‐148a, miRNA‐126,	Upregulated	Involved in apoptotic pathway. Triggers inflammation, exaggerated immunogenic response, and pathogenesis.	[77, 113, 114, 115]
	miRNA‐17, miRNA‐142‐3p, miRNA‐146a, miRNA‐125b	Downregulated		
Inflammatory bowel disorder	miRNA‐101, miRNA‐515‐5p, miRNA‐623, miRNA‐325, miRNA‐876‐5, miRNA‐1224‐5p, miRNA‐1226‐5p, miRNA‐1253, miRNA‐455, miRNA‐20a, miRNA‐17‐5p, miRNA‐424, miRNA‐16‐5p, miRNA‐21‐5p	Upregulated	Serve as biomarkers for disease diagnosis. Enhances paracellular permeability of the intestinal epithelium. Boosts zonulin expression and promotes epithelial permeability. Reduces the expression of aquaporin 3, resulting in a weakening of the intestinal barrier.	[116, 117, 118, 119, 120]
	miRNA‐24, miRNA‐107, miRNA‐10a, miRNA‐223, miRNA‐9, miRNA‐21, miRNA‐874, miRNA‐150, miRNA‐125b, miRNA‐17‐92,	Downregulated		

## 4 ROLE OF miRNA ON EPIGENETIC NETWORKS UNDERLYING AD PROGRESSION

Epigenetic parameters greatly influence cell signaling, differentiation, gene expression and morphogenesis of cellular development in an organism. Hence, they have an essential role in the onset of a disorder and its related genetic background.^121^ Various epigenetic factors are common in various disorders due to their long‐lasting effect on the nature and progression of the genetic regulation in a diseased condition. The mechanisms like DNA methylation and modification of histone proteins can contribute to the pathogenesis of various ADs.^122^ These epigenetic parameters can correspond to the initiation and prolongation of inflammation in ADs. It has been observed in various studies that miRNAs play a centric role as epigenetic regulators and prevent the progress of inflammation.^123^ Epigenetic regulation of miRNA is enhanced when regulated with different biomarkers that help in the subcellular level mechanism of the disease. The different inflammatory cytokines released that further facilitate the formation of autoantibodies interact with miRNA networks and consequently contribute to the pathogenesis of various autoimmune diseases.^8^ In recent studies, it has been found that circular RNAs (circRNAs), a class of noncoding RNA, have high stability and evolutionarily conserved properties.^124^ They are passive carriers of genetic expression and can affect auto‐antibody pathogenesis and formation. They act as noninvasive biomarkers and consequently act along with miRNA for its upregulation and downregulation. Thereby, it affects the progress of the disease synergistically.^125^ With the help of further computational analysis using the Kyoto Encyclopedia of Genes and Genomes (KEGG), scientists have inferred that the mRNA involved in the circRNA‐miRNA network is significantly involved in the secretion of various cytokines, which regulate various inflammatory pathways.^126^ CircRNA acts like miRNA sponges, which contribute to the development of various autoimmune diseases by various biological processes like DNA methylation and regulatory immune responses.^127^ The interplay and corresponding effects of the epigenetic regulation of miRNA is an area of ongoing research. Epigenetic therapies are a new area of clinical advancement that helps reverse epigenetic alteration or aberrations.^128^

### 4.1 RA

RA is a disease caused by loss of autoimmunity, that encompasses synovial fluid hyperplasia and inflammatory joint degradation. The inflammation in RA is mediated by several factors like ACPAs which further result in structural damage of the bone joints.^129^ The dynamic expression of the diseased condition in the patients is due to the epigenetic regulation of the genes directly associated with the immune system. The synovial fibroblasts consist of a unique methylation pattern, which varies during the course of gene progression and consequently affects its severity.^130^ L1 (LINE) is hypomethylated and indirectly induces the surge of cytokine, growth factors, receptor and inflammatory co factors.^131^ Local hyperinflammation spike is noticed in the RA patients due to the hypomethylation of the CpG islands associated with the IL6 promote gene. This leads to the overexpression of IL6 and related pro‐inflammatory cytokines.^132^ RF, anti‐carbamylated protein (anti‐CarP), and anti‐cyclic citrullinated peptide‐2 (anti‐CCP2) are the main autoantibodies that are responsible for the inflammation of multiple joints, during the progression of RA. hsa_circ_0038644 is spliced from protein kinase C beta gene, which is consequently related to the activation of NF‐κB. hsa_circ_0001859 would promote the expression of the transcription factors involved in the progression of the disease by targeting miRNA‐204.^133^ Various nuclear related factors (Nrf) were involved in the neuroprotection against oxidative stresses. These were affected by mRNAs like Atp6v0a1, Atp6v0b, Atp6v0c, and Atp6v0e2 which were then involved in the miRNA‐circRNA interaction network involved in the progression of the Nrf‐2 mediated development of RA.^134^ The effect of circRNA is extremely underlying and has far reaching effect on the progression of the disease. Recent research has verified that epigenetically regulated gene networks in the peripheral blood mononuclear cells consequently result in the severity regulation of RA. The synovial cells modify themselves in such a way that they start to show tolerance toward the apoptosis. This in turn triggers the inflammatory cascades in the pathogenesis pathway of RA development. This is majorly induced by the histone modification and hypermethylation of the NFkB promoter gene.^135^ The histone modifications like acetylation, methylation, citrullination, phosphorylation, and ubiquitination contribute to the changes in the transcriptional factors, making the chromatin available for gene expression. The different histones that take part in the RA pathogenesis and histone modification are—H3K9, H3K14, H4K5, and H4K16 (for acetylation), H2BK5, H3K4, H3K36, and H3K79 (for methylation), H3S10 and H3S28, H4S1 (for phosphorylation) and H2BK120 (for ubiquitination). These developments can in turn be used as biomarkers and contribute to the therapeutic application of this disease.^130^

### 4.2 Type 1 diabetes mellitus

Autoimmune dysfunction of pancreatic β cells leads to the development of type I diabetes. Its progression is monitored on a wider scale as it is widely affected by the epigenetic regulation of the various related genes.^136^ These epigenetic factors contribute in the glucose intolerance, due to the alteration in the methionine metabolism. The immunogenic response is seen to be related to the DNA methylation of several regulator genes. FOXP3 promoter region of the CD4+ is hypermethylated in the case of latent autoimmune diabetes in adults (LADA) patients.^137^ The INS gene promoter in diabetic patients is seen to be hypomethylated, which has consequently culminated in the release of inflammatory cytokines like TNF‐β, IFNγ, IL6, and IL‐1B.^138^ The histone modifications are mainly regulated by histone‐acetyltransferases (HATs) and histone‐deacetylases (HDACs), which regulate the process as transcription coactivators. RNA modification and ncRNA regulation are also noticed in the aged group of diabetic patients, which also attributes in cellular senescence and posttranscriptional modification.^139^ CircRNAs show participation in the onset and consequent development of type 1 diabetes melitus (T1DM). The downregulation of circRNAs 000286 and 017277 have a direct effect on the insulin synthesis and secretion.^140^ These further initiate apoptosis in pancreatic β cells and thereby induce the β dysfunction. They then further facilitate the immune system by affecting the production of macrophages which cause the loss of β cells and further induce hyperglycemia. hsa_circ_0060450, circPPM1F, and hsa_circ_0002202 are seen to be upregulated in peripheral blood mononuclear cells (PBMCs) in case of T1DM patients, that further help on the sponging of miRNA‐199a −5p that inhibits the JAK‐STAT signaling involved in the macrophage mediated inflammation.^141^ Various studies documented the histone modification of H3K9Ac, H4K16Ac, H3K4me3, H3K9me2, and 3, H3K27me3 genes in the diabetic pathway, further closely located to the DQB1 and DRB1 genes. This leads to a surge in the transcription process in the monocytic cell line, thereby triggering the immunogenic pathway.^142^ One of the most significant autoimmune markers of the progression type I diabetic condition is the reduced expression of the H3 histone acetylation that develops a consequent the expression of GAD autoantibodies.^143^

### 4.3 MS

MS is a neurodegenerative, inflammatory disease that results in physical and cognitive impairment. The progression of MS can be denoted by the degeneration of peripheral immune response to the growing immune processes in the central nervous system. This disease progression can be related to the various miRNA and noncoding RNA regulation at the subcellular levels.^144^ The metastasis associated lung adenocarcinoma transcript 1 (MALAT1) is a long noncoding RNA, the regulates the alternate splicing mechanism, which contributes to the abnormal RNA metabolism in MS patients. The circRNA formation is indirectly regulated by MALAT1 formation, which consequently interacts with various miRNA networks for the progression of the disease. The T cell exhibits autoreactivity that causes demyelination which consequently leads to an inflammatory cascade in the central nervous system. Studies have shown that the hypomethylation of promoter region peptidyl arginine deaminase (PAD)‐II contributes in the citrullination of myelin basic protein (MBP).^145^ This may lead to irreversible molecular changes that can cause insatiable and chronic inflammation. *ATXN1* gene which encodes for polyglutamine protein ataxin‐1, which in turn protects the system from the demyelination event. Studies have shown that in the event of hypomyelination of *ATXN1* gene, the pathogenetic mechanism scales up in the cellular and molecular regulation of MS. Thelper (Th) 17 and T regulatory (Treg) molecular balance has an important role to play in the progression and dynamics of MS, due to their epigenetic dynamic nature. Histone modification of forkhead Box P3 (FOXP3)‐ cell‐type‐specific regulatory regions (CSRs) and RAR related orphan receptor C (RORC)‐CSRs are polarized Th17 cells are regulated by estrogen in pregnant women suffering with MS, during their third trimester. A recent study shows the correlation of epigenetics and disease progression via epigenomic and transcriptomic profiling, where they have tried to compare demyelinated MS lesions and normal‐appearing white matter (NAWM). Human‐iPSC‐derived oligodendrocytes were epigenetically edited to understand the region‐dependent hypermethylation of MBP which is responsible for the myelination and axonal development.^146^ During the pathogenesis of MS, antibodies are formed against different histone proteins like H2b, H1, H3, H4, MBP, and DNA, that result in the hydrolysis of H2A histone. This cross reactivity between the abzymes and histone proteins can result in the aggravation of the disease condition.^147^ Pedre and colleagues reported the increased expression of H3 histone acetylation in chronic MS patients, which has also been seen as a side effect to in turn increase the transcription pattern of the inhibitors of oligodendrocytes and histone acetyltransferase (HAT) gene expression in MS patients.^148^

### 4.4 SLE

Autoantibodies and dysfunctional antigen presenting cells are the classical characteristics of SLE. The pathogenetic pathway of SLE is still a matter of debate, and has been seen to be largely affected by the epigenetic and environmental regulation over the patient's immune system. The multi organ pathologies and range of clinical manifestations involved in the progression of SLE is majorly immune related and still needs to be fully explored. Various circular RNA comprehensively is known to affect the progression of SLE. According to one study, hsa_circ_0012919 is found to abnormally upregulated in the T helper cells of SLE patients.^149^ The consequent downregulation of these circRNA can increase the expression of DNA methyl transferase1and reduce the expression of T cells in SLE patients. This regulation can further effect the expression KLF13, by synergistically acting along with miRNA‐125a‐3p. circIBTK bound with miRNA‐29b facilitates the inhibition of DNA methylation and further activation AKT pathway.^150^ According to Hedrich and colleagues, CpG‐DNA methylation patterns are highly conserved and close to the promoter region of IL7F. It is observed that the SLE patients show low degree of methylation in the T‐lymphocytes, as drawn parallels with the study on healthy individuals.^151^ In other recent findings, it has been seen that there is an increment in the levels of 5‐hydroxymethylxytosine (5‐hmC). This is due to the upregulation of ten eleven translocation (TET)−2 and TET‐3 factors, which are one of the prominent DNA methylases and contribute in the enzymatic conversion of 5‐methylcytosine (5‐mc) into 5‐hmC.^152^ In several SLE patients, DNA hydroxymethylation pattern is observed in the signaling pathway genes, which are then consequently related to the immune response genes and factors like SOCS1, NRF2F6, and IL15RA. The methylation product—5‐hmC and its aberrant regulation may contribute in the therapeutic application of SLE.^153^ The Th17 cell maturation takes place through the epigenetic modification if the transcriptional factors associated with it, in the SLE patients. The STAT3 pathway plays a pathbreaking role in the modification and modulation in T cell maturation and progression of SLE pathogenesis.^154^ Histone modifications in the SLE is reported in the form of trimethylation of histone H3 in H3K27me3, which contributes to the increase of H3K27me3 in the CD4+ T cells of the SLE patients. This trimethylation is made possible with the help of enhancer of zeste 2 polycomb repressive complex 2 subunits (Ezh2). The cumulative effect of these histone modifications may lead to the t cell lineage development in the SLE patients, which may attribute to the pathogenesis of the disease.^155^ Recent research and studies have shown and possibility of 3D genome alteration with respect to the progression of disease. The study included testing the viability of cells when subjected to histone modification of H3K27ac, H3K4me1, H3K4me3, SP11 knockdown, and transcription factor motif enhancement. These studies indicate the indirect effect of epigenetic regulation over the genome structure and function.^156^

### 4.5 IBD

IBD is an autoimmune, chronic, recurrent gastrointestinal disorder. Several factors like environmental factors, gut microbiota, immune dysregulation contribute to the advancement and cause of this disorder. Pathogenesis of this disease cannot be clearly elucidated as it can be manifested into colorectal cancer, fistula development or stenosis syndrome.^157^ Inflammatory bowel disease is an immune disorder where various pathogenic bacteria play a role in the maturation of the disease. There are various subcellular markers which can be used to detect the increased cytokines including Th17, IL1β1, and others.^158^ Circular miRNA has binding sites in various miRNA, where it causes the sponging of the miRNAs, which then consequently result in the progression of the inflammatory bowel syndrome. circRNA_004662 was seen to be highly expressed in the patients with IBD progression. circRNA‐102685 is found to be increased in the colon of IBD patients and is said to have affected the apoptosis and p53 regulation in the colon cells.^159^ This circRNA works as a sponging effect with miRNA‐146 that can interplay between the various immune cells. Mucosal methylation of HRAP2, FANCC, GBGT1, DOK2, and TNFSF4 in the progressive IBD, may cause a significant progression and severity of the disease.^160^ There are counter active findings which are noticed in the case of CD patients where GBGT1, IGFBP4, FAM10A4 genes are hypermethylated and in case of UC patients, IFITM1 is hypomethylated. This helps us to regulate and differentiate between the two subtypes. However, there is also the case of leukocyte methylation in the CD sub type of IBD.^161^ At the molecular level, most methylations occurring in the case of this disease, has a close proximity to GWAS risk genes, like CARD9, CDH1, ICAM3.^162^ According to a group of British scientists, the region of the gut that is more prone to DNA methylation of the intestinal region. Major histone modifications are found to be abundant in the following histone proteins—H2BK5ac, H3K36me1, H3K4me3, macroH2A, and Rme2sym, in the case of CD patients, as compared to the healthy people. These modifications can be consequently analyzed with the presence of natural killer (NK).^163^ This accounts for the epigenetic biomarkers for the progressive and detectable approach of IBD.^164^

## 5 DIAGNOSTIC AND BIOSENSING INTERVENTIONS USING miRNA AS A BIOMARKER FOR AD

**Figure 4:**
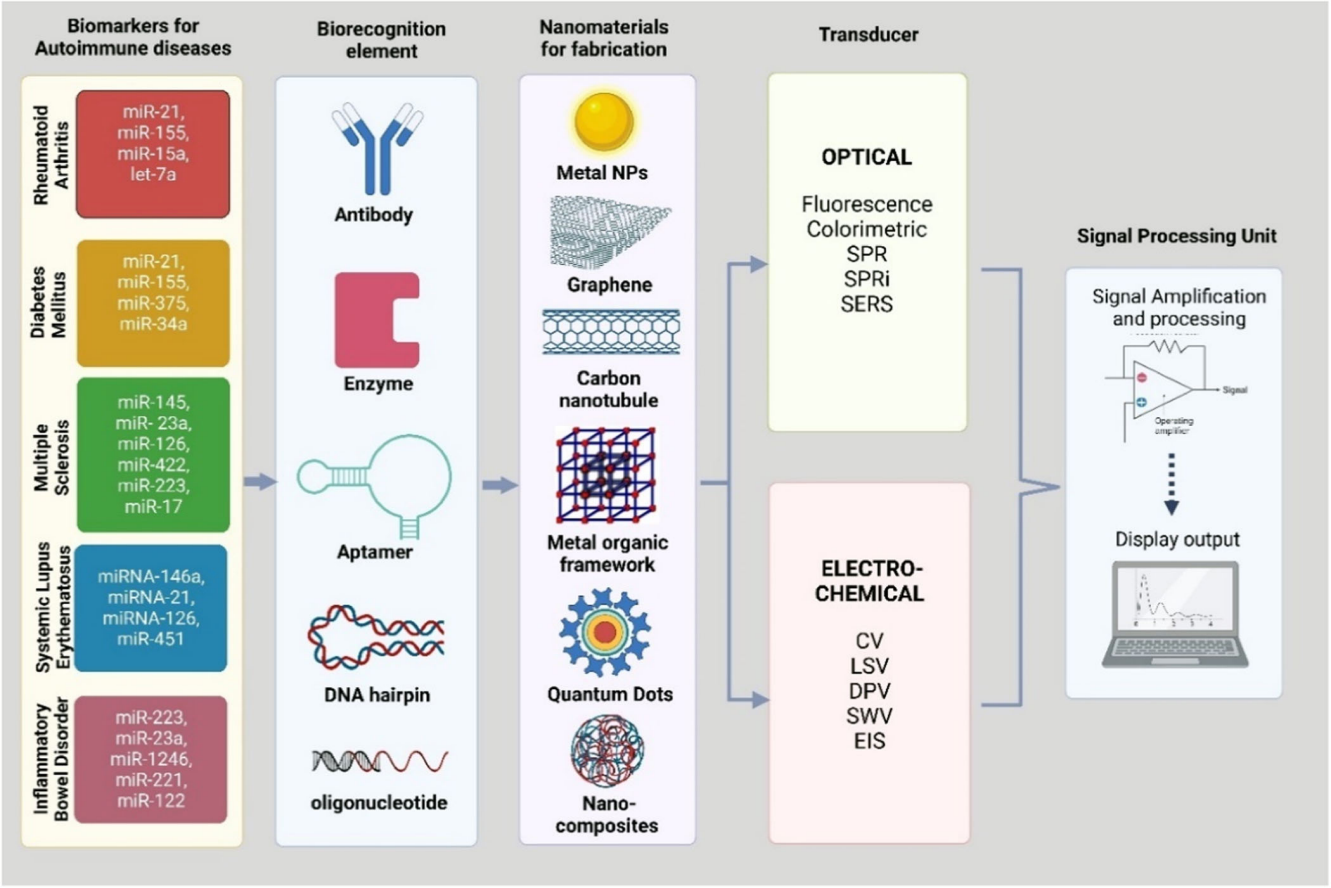
Illustrative depiction of biosensor construction deploying miRNA signature molecules corresponding to respective ADs using several biorecognition elements along with assistive nanomaterials for eliciting robust signal amplification to detect pathogenic miRNA indices. ADs, autoimmune diseases.

Globally, AD affects nearly 8% of the population, which includes approximately 80 disorders and exhibits several geo‐epidemiological variations, making it the fourth leading cause of mortality worldwide, after cancer and heart disease.^165^, ^166^ Women are 2.7 times more susceptible to developing AD as compared to men.^166^ Hence, rapid and early diagnosis of AD is gaining prime importance in improving the quality of patients' lives.^167^ Traditionally, diagnosing AD has been limited to detecting autoantibodies in patients' samples through western blotting, indirect immunofluorescence and ELISA‐based commercial assays. However, the pitfalls of such techniques include expensive antibodies, longer incubation time, the need for sophisticated instruments and complex procedures.^167^ A more sensitive and reliable technique is needed to diagnose AD due to limitations in accuracy and sensitivity,^167^ further summarized in Figure 4.

Recently, miRNAs have proven to be effective noninvasive biomarkers for the diagnosis and prognosis of a disease, to study its progression and to analyse the drug responses following treatment.^168^ This is because the abnormal miRNA expression profile is strongly correlated with diseased states, and research shows that they are present in almost all biofluids.^168^ Despite the advent of high‐throughput technologies, detecting miRNA comes with several predicaments. First, they are of short length (~15–25 nt), making it challenging to develop a highly specific probe. Second, distinct miRNAs harbor homologous sequences, prone to give false‐positive results due to cross‐hybridization.^169^ Conventional approaches detecting miRNA include qRT‐PCR, northern blotting, enzymatic assays, oligonucleotide microarrays, cloning, and sequencing, which come with several drawbacks such as limited selectivity, low sensitivity, and ineffectiveness in detecting deficient miRNA concentrations in blood samples.^170^ All these molecular techniques lack an integrated transducer element and are amplification‐based.^171^ In addition to being time‐consuming and laborious, these techniques need to consider the susceptibility of miRNA to degradation, giving rise to biased, unreliable results.^171^ Furthermore, these detection techniques do not encompass multiplexed analysis, in vivo analysis, detecting circulating miRNAs and specificity toward single‐nucleotide, all of which are indispensable in clinical settings.^169^

With the breakthrough of nanotechnology in medicine, amplification‐free biosensors have drawn considerable attention from academia and the industry.^172^ A biosensor is an integrated analytical device consisting of three components: (i) a bio‐recognition element such as enzymes, antibodies, DNA, RNA, aptamers, and so forth, (ii) a transducer element which detects a biological response and converts it into an electrical signal and (iii) a signal‐ processing system that consists of an amplifier, a processor and a display unit.^172^ This analytical device is highly effective in quantitative or semiquantitative detection of an analyte.^167^ It incorporates specific DNA probes, complementary sequences with target miRNA, and a highly adaptable transducer sensing system.^171^ This enhances their multiplexing potential, allowing them to provide rapid, label‐free, extremely selective, and sensitive real‐time detection of miRNA concerning point‐of‐care (POC) aims for clinical applications.^169^ Based on the type of signal transducers used, biosensors for detecting miRNA are broadly classified into optical and electrochemical biosensors.^167^ Optical biosensors are highly effective in detecting biomarkers as they transduce an optically active reporter's absorbance or fluorescence signals linked to a nucleic acid probe when hybridized to the target miRNA.^169^

This biosensor class has a relatively simple and feasible design, wherein the bio‐recognition elements are immobilized on the surface of a signal detection platform by either physical adsorption, covalent or electrostatic bonding.^168^ The detection platforms are fabricated with nanomaterials such as graphene oxide (GO), gold nanoparticles (AuNP), and quantum dots (QD). AuNPs are highly compatible with nucleic acids and proteins, provide adequate surface area to volume ratio, have excellent fluorophore quenching ability and exhibit good LSPR absorption in the visible range.^168^ Surface Plasmon resonance (SPR) is another robust and sophisticated optical method for fast and direct miRNA detection,^173^ measures the shifts in refractive index when an analyte forms a complex on the surface of the electrode.^169^ Surface plasmon resonance Imaging (SPRi) is a label free hybridization‐based method and can measure miRNA concentration for as low as 2 pM in less than 30 min.^170^ Their limit of detection (LOD) is low as far as complex biological samples are concerned.^169^

Several strategies are employed to enhance its sensitivity, such as using nanoparticles like AuNPs coupled with DNA sandwich, GO–AuNPs hybrids, hybridization chain reaction method, hairpin assembly, streptavidin‐biotin approach, and so forth.^169^ Surface‐enhanced Raman scattering (SERS) is employable for rapid and accurate miRNA detection but is unsuitable for medical diagnostics due to low sensitivity.^173^ Fluorescence detection is a biosensing strategy that uses optical methods. It includes several examples, such as Ag nano‐cluster DNA probes, GO with dye‐labeled probes, carbon nanoparticles, and magnetic beads.^169^ Polyaniline‐gold (PANI‐Au) nanomaterial and ruthenium (Ru)‐based electrochemiluminescence immunosensors have also been designed for label‐free, highly sensitive quantification of miRNA.^167^ SPR‐based biosensors are commonly employed optical methods for diagnosing AD due to their high selectivity, high efficacy, affordability, and reliability. However, biosensors working on the principle of electrochemiluminescence demonstrate the highest sensitivity. Electrochemical biosensors are the most predominantly used sensors for the diagnosis of AD.^167^ The transducer is a solid electrode sensitive to changes in electrode properties caused by the hybridization between the immobilized nucleotide probe and the complementary sequence.^170^ In these, the transduction element is often Au, indium tin oxide, glassy carbon, and graphite, the sensitivity of which is enhanced by incorporating a variety of nanoparticles, nanowires and enzymes.^169^ In contrast to optical biosensors, electrochemical biosensors have simpler electronic designs, are inexpensive, and serve as excellent platforms for point‐of‐care tests because of their easy‐to‐use miniaturized portable integrated systems.^173^

The amperometric and voltametric techniques measure changes in the current when a target miRNA is hybridized to its complementary sequence, with the only difference being that the current is measured at a fixed potential value in amperometry.^169^ Cyclic voltammetry (CV), linear sweep voltammetry (LSV), differential pulse voltammetry (DPV), and square wave voltammetry (SWV) are different techniques under voltammetry.^167^ These are further categorized as label‐free biosensors if the redox reaction is generated due to an electroactive nucleic acid base (adenine or guanine) signals or enzymes such as duplex‐specific nuclease; or as label‐based biosensors if the electrochemically active reporter species are nanoparticles (e.g., Au, Ag, OsO2, or Ruthenium NPs) or hybrid nanoparticles (e.g., GO–AuNPs, MoS2 microcubes, Au@NPFe2O3 nanocubes. Electrochemical impedance spectroscopy (EIS) is a well‐established label‐free technique for quantifying miRNA. It is practical for studying reaction mechanisms, biofunctionalization, nanostructure formation, and hybridization.^169^

Both optical and electrochemical methods have pros and cons in detecting miRNA, with some giving a much lower detection limit and, hence, higher sensitivity than others. However, selecting an optical or an electrochemical biosensor would depend upon the experimental aim, which would differ for research and clinical purposes. In conclusion, biosensors offer unique advantages over traditional assays, making them highly suitable POC devices for diagnosing AD. The incorporation of nanoparticles in these biosensors has significantly brought down the LOD to pM and fM range, which is instrumental in the rapid and early‐stage diagnosis of AD. Further innovation is needed to develop in vivo sensing platforms for AD.^167^ Table 2 lists down the different optical and electrochemical techniques used for detecting miRNAs in RA, Diabetes, MS, SLE and IBD, along with their references.

**Table 2:** Different techniques, their range and mechanism of detection of miRNAs in different autoimmune diseases.

microRNA	Optical technique	Linear dynamic range	Disease	Mechanism	References
let‐7a	Fluorescence	10 fM–2 pM	RA	Helicase (RecQE) ‐assisted hybridization chain reaction on graphene oxide (GO) platform	[174]
miRNA‐155	Absorbance	100 aM–100 fM	RA	DNA probe bound to citrate‐capped Au nanoparticles; target miRNA adsorbed on polyethylenimine‐capped AuNP surface	[175]
miRNA‐15a	Surface plasmon resonance (SPR)	5 fM–0.5 nM	RA	Isolated Au islands on innovative chip installed on SPRi imager bordered by hydrophobic fluoropolymer‐ CYTOP	[176]
miRNA‐21, miRNA‐155	Surface plasmon resonance (SPR)	10 aM–10 pM	RA	SPR chip surface coated with antimonene nanosheets; amplification due to interaction with AuNR‐ssDNA	[177]
miRNA‐155	Surface enhanced raman spectroscopy (SERS)	1 fM–10 nM	RA	SERS enhanced DSN amplification in DNA microcapsule using TB@CaCO3 blend	[178]
miRNA‐145	Fluorescence spectrophotometry	N/A	MS	Hybridization chain reaction‐based formation of AgNCs in DNA (highly fluorescent)/use of oligonucleotide hairpin probes	[179]
miRNA‐ 23a, miRNA‐126, miRNA‐422, miRNA‐223	SPRi	N/A	MS	Enzyme‐free SPR‐based nanoenhancer composed of neutravidin‐coated gold nanospheres (nGNSs); miRNA recognition through use of antibody against DNA/RNA hybrids	[180]
miRNA‐17	LSPR	N/A	MS	Immobilized AuNPs on aminopropyl triethoxysilane (APTES)‐treated glass slides; amplification based on HCR and hairpin surface‐tethered probes	[181]
miRNA‐155	Colorimetric‐based	100 aM–100 fM	MS	DNA probe bound to citrate‐capped Au nanoparticles; target miRNA adsorbed on polyethylenimine‐capped AuNP surface	[175]
miRNA‐155	cyclic voltammetry (CS), electrochemical impedance spectroscopy (EIS)	10 aM–1 μM	MS	Bioreceptor attached to single‐walled carbon nanotubes (SWCNT) and polypyrrole (PPY) nanocomposite on graphite sheet platform followed by hybridization with target miRNA	[54]
miRNA‐146a	LSPR	‐	SLE	Detection of extracellular vesicles using plasmonic nanoparticle‐embedded polydopamine platform	[182]
miRNA‐21	photoelectrochemical	0.01 fM–1 μM	SLE	ZnIn_2_S_4_ QDs heteroconjugated with TiO2 signal probe; enzyme‐free target cycle amplification using tripod DNA walker	[183]
miRNA‐126	electrochemical	1 fM–10 nM	SLE	Target miRNA immobilized on CoNi‐MOF metallic framework using 2,2ʹ‐bipyridine‐5,5ʹ‐dicarboxylic acid as ligand	[184]
miRNA‐451	(LC–MS) bioanalytical method	0.5–200 ng/mL	SLE	Biotinylated capture strands and NEB hydrophilic streptavidin magnetic beads	[185]
miRNA‐223	Fluorescencespectrophotometry	0.05–0.6 μM	IBD	cDNA probe (P1–4) paired against target miRNA to quench fluorescent DNA/AgNC moiety; based on fluorescence turn‐on strategy	[186]
miRNA‐23a and miRNA‐223	Fluorescencespectrophotometry	0.05–0.8 μM	IBD	Multicolored fluorescent DNA‐stabilized AgNCs/two split DNA probes	[187]
miRNA‐1246, miRNA‐375, miRNA‐21, and miRNA‐221	Electrochemical biosensor	10 fM–100 pM	IBD	Exo‐miRNA analysis using DNA‐tetrahedrons‐assisted catalytic hairpin assembly (MDTs‐CHA)	[188]
miRNA‐375	Label‐free electrochemical biosensor	10 and 30 fM	IBD	Oligonucleotide capture probe immobilized on Au electrode, followed by hybridization	[189]
miRNA‐122	Electrochemical DNA sensor	5 pM–10 nM	IBD	Toehold‐promoted strand displacement reaction in presence of miRNA + Exo (III) followed by enzymatic cyclic amplification reactions	[190]

Abbreviations: IBD, inflammatory bowel disorder; MS, multiple sclerosis; SLE, systemic lupus erythematosus.

## 6 miRNA BASED TARGETED THERAPEUTICS IN ADs

**Figure 5:**
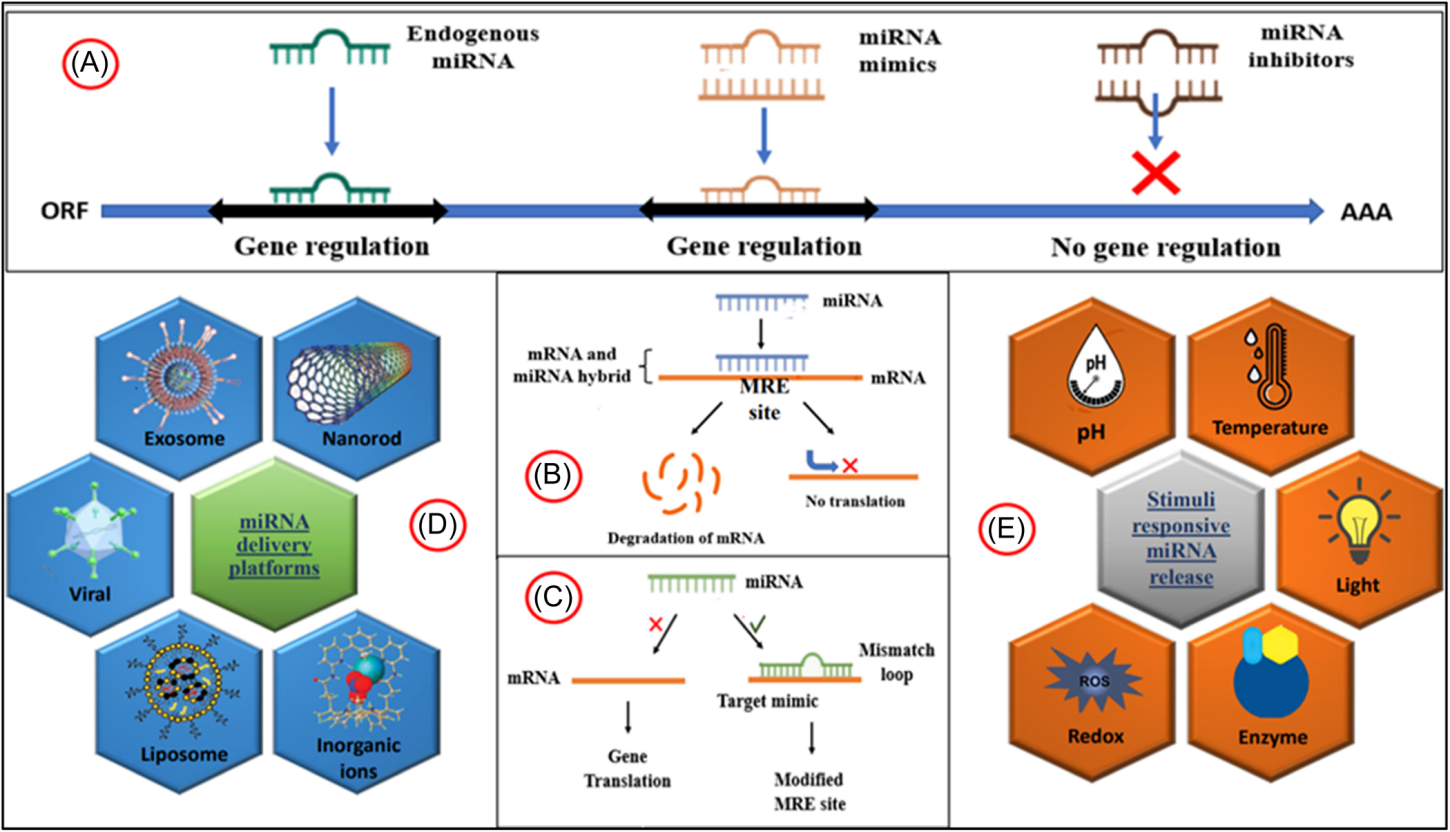
Representative demonstration of miRNA‐based and delivery of miRNA signatures through various nonviral and viral vectors. (A) Comparative regulatory summarization of endogenous miRNA, miRNA mimics and miRNA inhibitors on gene expression. (B and C) Molecular mechanisms concerning an overview on miRNA silencing and mimicry for controlled gene expression. (D) Several innovative miRNA delivery platforms for therapeutic alleviation of different ADs. (E) Recent advanced miRNA delivery platforms sensitive to internal or external stimuli. ADs, autoimmune diseases.

The two main branches of miRNA‐based therapeutics of AD are mimics and anti‐miRNAs/inhibitors. Mimics are artificial double‐stranded small RNA analogs to the corresponding miRNA. Inhibitors are single‐stranded, and they target miRNAs.^191^ The first miRNA therapeutic drug was miravirsen, a short antisense RNA oligonucleotide complementary to the miRNA‐122. It is used to treat infection caused by the hepatitis C virus in phase II of clinical trials.^192^ Several studies have briefed the higher induction of B‐and T‐cells responses and specificity via miRNAs compared to other nucleic acid‐based conventional vaccines. But, the shorter half‐life in the transient, controlled expression of encoded antigen, the risk of genomic integration or mutagenesis,^193^ degradation,^194^ maximum delivery to the target cells, and so forth,^195^, ^196^, ^197^ have simultaneously expanded the interest in the scientist to invent viral or nonviral based delivery of miRNAs to overcome the challenges.^198^ The clinical outcome of viral gene therapy established enormous interest in the scientists, but at the same time, confronting many more additional challenges, such as pre‐existing immunity, undesirable genomic integration, viral‐induced immunogenicity, ineptitude to re‐dose, difficulties involved in upscaling, payload size constraints, and so forth.^195^ Furthermore, to confound these challenges, scientists have been researching for the last 15 years to develop nonviral‐based miRNA delivery, that is, delivery encapsulated via polymeric, lipid‐based, inorganic/metallic‐based, stimuli‐responsive, exosome‐mediated delivery systems for various ADs and have established the successful clinical readouts, supported from Food Drug Administration (FDA) even for the administration of LNP‐based mRNA COVID vaccines.^195^ The delivery of miRNAs via various nonviral vectors have been summarized in Figure 5.

RA is the most common systemic autoimmune joint inflammatory disease due to proteomic, epigenetic, and genetic factors. RA miRNAs (~22 nucleotides) categorized under epigenetic factors, further apt of gene expression modulation, have been concluded as biomarkers for diagnosis, prognosis, and response to treatment in RA.^199^ The miRNA‐124 is an endogenous noncoding RNAs identified for therapeutic potential via blocking NF‐kB ligand‐based receptor activator (RANKL) and T cell cytoplasmic 1 (NFATc1) in RA. Therefore, Zhao and colleagues, developed ketoprofen and miRNA‐124 co‐loaded in poly (cyclohexane‐1,4‐diylacetone dimethylene ketal) (PCADK) nanoparticles (NPs) via emulsified solvent evaporation method. The study resulted in NPs of 160 nm of hydrodynamic diameter, stability at 37°C, and showed higher therapeutic effect within Day 17 with no significant side effects and neutral pH of the PCADK. Thus, the study showed that acid‐sensitive NPs are an optimistic approach to systemic RA inflammation.^200^ The conventional treatment strategies in bone erosion, especially in RA, have exhibited low disease specificity with higher recurrence. Hence, Sujitha and colleagues, developed PEGylated lipid‐based nanoparticles loaded with miRNA‐23a/berberine (BBR) for the treatment of Wnt1/β‐catenin mediated bone erosion in adjuvant‐induced arthritic (AA) rat model. The study resulted in higher expression levels of β‐catenin, FZD4, Dvl‐1, and LRP5 through the induction of cylindromatosis (CYLD), which further enriched calcium concentration by binding Osteoprotegerin (OPG) with receptor activator of nuclear factor kappa beta (NFkB ligand). Therefore, this study documented the potentiality of miRNA‐23a for targeting Wnt1/β‐catenin signaling in RA disease.^201^ The macrophages recreate a climacteric role in the polarization of M1 or M2 phenotypes under a stimuli‐responsive microenvironment in bone healing. Recently, Li and colleagues, characterized miRNA‐based nanocarrier synthesized via free radical polymerization and reported higher cellular uptake and sequential delivery of miRNA‐155 and miRNA‐21 for the timely polarization of M1 to M2 in the treatment of bone tissue engineering.^202^ To control the inflammation in RA, Deng and colleagues, investigated miRNA‐21 and IL‐4 loaded α‐helical polypeptide nanocomplexes under acidic‐stimuli microenvironment for RA. The miRNA‐21/IL‐4 attenuates negative to positive in inflamed synovium, directing to macrophage polarization, promoting tissue repair, NF‐κB inhibition, and so forth. Thus, this unique delivery system for exosomes or genes could be further persuasive in RA treatment.^203^ The synovial tissue inflammation and joint destruction associated angiogenesis is the main focus in the treatment of RA. In this context, to deliver genetic information or microRNAs between cells to restrict the inflammation, exosomes play an important role. Therefore, Chen and colleagues, investigated the mesenchymal stem cell (MSC) derived miR‐150‐5p loaded exosome (Exo‐150) in RA to assess the MMP‐14, fibroblast‐like synoviocytes (FLS), and vascular endothelial growth factor (VEGF) in RA patients via ELISA, and western blot analysis. The in vivo study resulted Exo‐150 exhibited the downregulation of HUVECs by targeting MMP14 and VEGF, reduced paw thickness in collagen induced arthritic mice, further inhibiting synoviocyte hyperplasia and angiogenesis.^204^ In a recent year, Islam and colleagues, investigated case control analysis of the association between miRNA‐146a and miRNA‐499 in RA patients from Pakistan. A noteworthy correlation between the genotypes of miRNA‐146a and miRNA‐499 and patients diagnosed with RA was identified. The miRNA‐146a rs2910164 G allele and the miRNA‐499 rs3746444 C allele are significantly associated with RA compared to the control group. Additionally, the transmission analysis demonstrated a significant hereditary transmission of the rs2910164 G allele and rs3746444 C allele from parents to affected offspring. The present study's findings indicate an association between miRNA‐146a (rs2910164; C>G) and miRNA‐499 (rs3746444; T>C) polymorphisms with RA among the examined population. Moreover, our study revealed, for the first time in our high‐risk population, a significant association between the rs2910164 G allele and the rs3746444 C allele and familial RA.^205^

Type‐I diabetes (TID) is the most common AD, leading to the selective destruction of islet β cells by the body's immune cells. Therefore, miRNAs such as miRNA‐142‐3p, miRNA‐142‐5p, and miRNA‐155 from human T lymphocyte exosomes have been deployed to form β cells favoring apoptosis, cellular differentiation, and cell growth.^206^ Furthermore, Guay and colleagues, reported exosome‐mediated apoptosis, higher insulin level and reduced inflammation in nonobese diabetic (NOD) mice via T lymphocytes mediated apoptosis and chemokines signaling. Thus, the study resulted in miRNA‐exosomal delivery to promote the communication between immune and islet β cells.^206^ The miRNA‐30a, predominantly suppressed by the Notch signaling pathway in the hyperglycemic kidney, further impacted podocytes in diabetic nephropathic conditions. Thus, to deliver exogenous miRNA‐30a, Raval and colleagues, developed miRNA‐30a based cyclo (RGDfC)‐gated polymeric‐nanoplexes with dendrimer nanostructure to improve podocyte conditions. The in vivo study showed the suppression of Notch‐1 in streptozotocin (STZ) C57BL/6 mice, followed by the higher expression level of miRNA‐30a, reduced glomerular expansion, and fibrosis. Thus, the nanoplex system could be utilized clinically for exogenous miRNA delivery in kidney‐related diseases.^207^ To improve the delivery efficiency of miRNA, Moraes and colleagues, embedded miRNA in genipin crosslinked nanogels (G‐PECs) via electrostatic interactions. The in vitro study resulted in 94.56% cytocompatibility of G‐PECs within human endothelial cells after incubation (01 Day) with a hemolysis rate of 2.09%, substantiating the nanogels as an effective nano platform for miRNA‐based delivery system in atherothrombotic‐related diseases.^208^ To assess the efficacy of miRNA in addressing erectile dysfunction (ED) generated by T1D, Tang and colleagues conducted a study examining the impact of miRNA‐92a on ED in rats with streptozotocin‐induced T1D. This investigation involved histological analysis of penile cavernous tissues. The study yielded findings indicating heightened activity within the eNOS/NO/cGMP signaling pathway, enhanced proliferation of cavernous endothelial cells, elevated expression of endothelial cell‐cell junction proteins, and reduced levels of oxidative stress. The expression of miR‐92a was seen to exhibit a notable rise in endothelial cells that were subjected to high glucose treatment. This increase in miR‐92a expression impeded the AMPK/eNOS and AMPK/Nrf2/HO‐1 signaling pathways in rat aortic endothelial cells by targeting Prkaa2. Consequently, this disruption led to endothelial dysfunction and an excessive state of oxidative stress. Therefore, miRNA has shown inhibitory effects on oxidative stress and endothelial dysfunction, leading to an improvement in diabetic ED.^107^

The immune system imbalance further impacts the CNS, which plays a vital role in the prognosis of MS, an AD. The miRNAs involved in the pathogenesis of MS influence the peripheral immune and glial cells and are also accountable for the differentiation, proliferation, and apoptosis of various cells.^209^ The higher stability and specificity of miRNA‐219 in CNS delivery assemble them an ideal candidate for MS via conjugating with tragacanthic acid (TA)/chitosan (CS)/glutathione (Glu) polyplex nanoparticles. The cuprizone model of MS mice, upon injecting the polyplex NPs, showed proteolipid protein 1 (Plp1) overexpression leads to reduced inflammation, apolipoprotein E downregulation, crystallin alpha B upregulation, and improved myelin sheaths in the brain. Therefore, glutathione‐targeted Ch/TA nanoparticles could be exploited as a feasible nonviral vector for miRNA‐219‐specific targeting to the brain for inflammatory abatement in MS.^210^ The regeneration of myelin (called remyelination) is the key strategy to combat MS in the brain. To promote remyelination via miRNAs, Osorio‐Querejeta and colleagues, investigated miRNA‐219a‐5p embedded Distearoylphosphatidylcholine (DSPC) liposome and poly‐lactic‐co‐glycolic acid (PLGA) nanoparticles in C57BL/6 female MS mice. The study resulted in higher encapsulation, release, and remyelination promotion overexpression of miRNA‐219a‐5p, leading to the highest oligodendrocyte precursor differentiation levels by extracellular vesicles. Therefore, the study concluded as promising strategy to deliver miRNA‐219a‐5p to induce remyelination in MS patients.^211^ Several research findings have demonstrated that the utilization of bone marrow mesenchymal stem cells (BMSCs) exhibits promising therapeutic capabilities in the management of MS.^110^, ^211^, ^212^, ^213^ The BMSCs can secrete extracellular vesicles known as exosomes (BMSC‐Exos), which encapsulate bioactive molecules with potential therapeutic effects for MS. A recent investigation conducted by Fan and colleagues, examined the underlying mechanism of bone marrow‐derived mesenchymal stem cell‐derived exosomes (BMSC‐Exos) containing miR‐367–3p in BV2 microglia and a murine model of multiple sclerosis known as experimental autoimmune encephalomyelitis (EAE). The findings of the study revealed that miR‐367–3p exhibited binding affinity towards Enhancer of zeste homolog 2 (EZH2), resulting in the upregulation of solute carrier family 7 members 11 (SLC7A11). The upregulation of SLC7A11 resulted in the activation of Glutathione Peroxidase 4 (GPX4), leading to the inhibition of ferroptosis, a form of programmed cell death. The research conducted demonstrated that the utilization of BMSC‐Exos containing miR‐367–3p resulted in a decrease in ferroptosis incidence within microglia. Consequently, this intervention exhibited a mitigating effect on the severity of experimental autoimmune encephalomyelitis (EAE) in an in vivo setting. The results of this study indicate that the upregulation of miR‐367–3p may hold significant potential as a therapeutic approach for MS.^214^

SLE corresponds to another segment of AD leading to deteriorating morbidity and quality of life, demanding immediate epigenetic solutions. The exosomal miRNAs can directly interact with Toll‐like receptors signaling pathways and regulate the activation of NF‐κB and secretion of inflammatory cytokines.^215^ The overexpression and inhibition of the associated miRNA have the common therapeutic effects of reduced expression levels of pro‐inflammatory cytokines, inhibition of autoantibody production, mitigated B cell activation, proliferation, decreased CD4/CD8 T cell ratio, reduced Fas receptor‐expressing lymphocytes, inhibited IL‐17, TNF‐α, or IL‐1β–induced NF‐κB activation and inflammation, enhance the immunosuppressive capacity of Treg cells, Inhibited abnormal Th17 cell differentiation, inhibited glomerular cell proliferation, reduced fibrinoid necrosis and fibrosis in the kidney, decreased proteinuria, and reduced anti‐dsDNA antibodies.^216^ The miRNA‐155 plays a fundamental part in evolving Diffuse alveolar hemorrhage (DAH) condition, that is, a painful complication due to SLE, in pristane‐induced lupus treated via miRNA‐155 antagomir. The study reported a reduction in the expression of pro‐inflammatory cytokines, NF‐κB signaling, inhibition of peroxisome proliferator–activated receptor α, etc. Furthermore, miRNA‐155 antagomir could be further ascertained as a promising therapeutic in inflamed lupus lungs.^217^ The limitations associated with glucocorticoids (GCs) in treating SLE have shown interest in developing novel miRNAs‐based nanoplatforms to target T cells and hyperactive B cells.^218^ In a recent year, Zhang and colleagues, constructed miRNA‐125a embedded monomethoxy (polyethylene glycol)‐poly(d,l‐lactide‐co‐glycolide)‐poly(l‐lysine) (mPEG‐PLGA‐PLL) nano platform (PEALmiRNA‐125a) to target splenic T cells. The in vivo study improved biocompatibility, circulatory time, and protection of miRNA‐125a from degradation into the splenic T cells in the SLE mice model and exhibited the efficiency of PEALmiRNA‐125a in SLE treatment.^219^ SLE is distinguished by an excessive response of B cells and the continuous production of autoantibodies capable of causing harm to multiple organs and tissues. Recent research findings have indicated that the suppression of miR‐7 through antagomiR‐7 can mitigate B cell hyperresponsiveness and serve as a preventive measure against the development of lupus. To exploit this advantage, Guo and colleagues, devised a nano delivery system using SA (sialic acid)‐poly (D, l‐lactide‐co‐glycolide) (SA‐PLGA) to encapsulate antagomiR‐7 (SA‐PLGA@antagomiR‐7) for targeted distribution into splenic B cells. This approach was chosen due to the persistent obstacles associated with the stability and precise delivery of miRNA in vivo. The research findings indicate that SA‐PLGA@antagomiR‐7 possesses favorable biocompatibility and protects antagomiR‐7 against degradation, hence prolonging the presence of the miRNA in the circulatory system in vivo. The therapy reduces immunological abnormalities, restores normal splenic B cell subtypes, and inhibits B cell activation.^220^

The delivery of miRNAs via nanoplatforms in inflammatory diseases such as IBD could show promising responses to TNF‐α expression. Recently, Louiselle and colleagues, conjugated cerium oxide nanoparticle (CNP)/miRNA‐146a embedded chitosan‐based gel to target the unregulated inflammatory responses witnessed in IBD/colitis. The authors hypothesized that the chitosan‐based gel reduced TNF‐α expression upon acquiring a single Chitosan‐CNP‐miRNA‐146a in dextran sodium sulfate (DSS)‐murine model of colitis/IBD. Thus, these nanoplatforms could be further clinically investigated for colitis/IBD due to less sensitization and more reasonable systemic effect than conventionally administered drugs targeting TNF‐α.^221^ Furthermore, to comprehend the clinical outcome of plant‐derived miRNAs in IBD, Zhang and colleagues, characterize the specific population via edible ginger‐based NPs (GDNPs) upon oral administration in colon targeting. The mouse colitis model showed reduced acute colitis‐related cancer, enhanced intestinal repairment by inducing pro‐inflammatory cytokines, and improved interleukins via GDNPs expressing ∼125 miRNAs with high lipids, proteins, and so forth. Thus, GDNPs show a natural delivery mechanism of miRNAs for improving IBD with no significant potential toxicity corresponding to synthetic NPs.^222^ Recent advancement reveals that Exosomes derived from human umbilical cord mesenchymal stem cell (hucMSC‐Ex) alleviates IBD in mice. The overexpression of miRNA‐302d‐3p in hucMSC‐Ex resulted in lymph angiogenesis via tyrosine kinase 4 (FLT4), reduction of vascular endothelial growth factor receptor 3 (VEGFR3), and infiltration of macrophages. Thus, the Exosome nano platform further regulates lymph angiogenesis via the miRNA‐302d‐3p/VEGFR3/AKT to ameliorate IBD.^223^

## 7 CONCLUSION AND FUTURE OUTLOOK

The human immune system is a complex network of cells with a variety of functions that collectively act to fight off infections, eradicate pre‐cancerous cells, and preserve metabolic health. Small genetic and epigenetic changes in the immune system can be deadly for some people. Even minor alterations in its ability to distinguish between the body and foreign invaders can result in various autoimmune diseases. Immune system disorders, with an increasing impact on the world's population, have become a significant socioeconomic problem on a global scale. ADs have critical and growing unmet clinical requirements because the majority of current medicines function widely and are not disease‐specific; hence, as a result, they are responsible for causing a variety of side effects. As a result, there is an urgent need for the production of novel medications or the repositioning of existing ones based on clinical and biochemical knowledge of a particular AD in individual patients. Over the past decade, there has been a significant improvement in understanding RNA's diverse physiological roles for medical applications. Numerous types of RNA have regulatory functions in tissues and cells; hence, these RNAs have the potential for modern therapeutics, with RNA acting as a target or drug.

miRNAs act as regulatory checkpoints and can behave as potential medicines, biomarkers for diagnosing diseases, or potential targets for other RNAs. Other potential RNA types that can be used as future targets or therapeutics are exosomal RNA, shRNA, circRNA, lncRNA, and saRNA. The treatment of ADs like RA is done by suppressing TNF, IL‐6, IL‐17, and IL‐23, and the treatment of SLE by inhibiting B cell survival factors is now approved. Targeting co‐stimulatory molecules and RNAi pathways can modify T cell activity as potential AD therapeutics. The stability and specificity of RNAi‐based techniques can be improved by the optimal design of shRNA, miRNA, and siRNA. Most ADs exhibit modified miRNA levels, and cytokine expression contributes significantly to autoimmunity's pathogenicity. Therefore, cytokine neutralization can be used as a therapeutic approach toward treating Ads. Due to the advent of rigorous scientific expeditions in this domain, an optimistic insight has been obtained on the effect of miRNA regulation and dysregulation associated with the onset or prevention of ADs and autoimmunity.

Despite the numerous and potential application of miRNA as a biomarker in autoimmune diseases, there are some discrepancies that annihilate the usage of miRNAs to its complete potential.^224^ One of the major concerns with the miRNA usage is the lack of consensus in the preparation of miRNA that are to use in clinical practice.^225^ miRNA profiling and technical aspects regarding the isolation procedure of the same is not clearly resolved and lacks internal controls. The miRNA agents fail to attribute their stipulated effect in the in vivo scenario.^226^ Furthermore, the drug delivery in miRNA therapy is extremely challenging. The miRNA drug is easily degraded in the first pass metabolism due to the action of nuclease on the miRNA. A possible concern may arise if the miRNA is used in a higher dose to surpass the degradation quotient, leading to unwanted side effects.^227^ They may cause cross reactivity in various pathologies or may aggravate a particular stage of a disease. The miRNA biomarker detection and reproducibility are a cumbersome process. Specificity and sensitivity of every molecular assay regarding the detection of RNA is highly variable, which makes the regularization of the assay very difficult.^228^ Various bioinformatic platforms and algorithms have been made for the regulation of miRNA target prediction that dysregulated the streamline nature of miRNA research and prediction of different binding sites of the miRNAs.^229^ Hence, there is ardent need for novel methodologies that can be used for the accurate and quantitative analysis of the miRNA used as biomarker or drug in miRNA therapy. There are unique ways to treat or prevent ADs that involve using miRNAs as a possible tool or target. By creating personalized drugs and using new diagnostic and prognostic methods, clinicians can gain a deeper understanding of the disease and perform clinical tests tailored to individual patients, considering differences in ethnicity and patient outcomes. This approach can improve current therapies and show how future research, technology advancements, and clinical tests can work together to understand the disease better and develop more effective treatments.

## AUTHOR CONTRIBUTIONS

**Sagnik Nag**: Conceptualization; writing—original draft; data curation; validation; formal analysis; writing—review and editing; visualization; validation; supervision; project administration. **Oishi Mitra**: Writing—original draft; data curation; validation; formal analysis; writing—review and editing. **Garima Tripathi**: Writing—original draft. **Souvik Samanta**: Writing—original draft; visualization; validation. **Bikramjit Bhattacharya**: Writing—original draft. **Priti Chandane**: Writing—original draft. **Sourav Mohanto**: Writing—original draft; data curation; validation; formal analysis; visualization; validation. **Vino Sundararajan**: Writing—original draft; data curation; validation; formal analysis; supervision; project administration. **Sumira Malik**: Writing—review and editing. **Ranjit Sah**: Writing—review and editing; supervision; project administration. **Joshuan J. Barboza**: Writing—review and editing. **Sarvesh Rustagi**: Writing—review and editing. **Suraj Adhikari**: Writing—review and editing. **Aroop Mohanty**: Writing—review and editing. **Darwin A. León‐Figueroa**: Writing—review and editing; supervision; project administration. **Alfonso J. Rodriguez‐Morales**: Writing—review and editing; supervision; project administration. All authors have read and agreed to the published version of the manuscript.

## CONFLICT OF INTEREST STATEMENT

The authors declare no conflict of interest.
